# Quadratic unconstrained binary optimization and constraint programming approaches for lattice-based cyclic peptide docking

**DOI:** 10.1038/s41598-025-05565-1

**Published:** 2025-07-01

**Authors:** J. Kyle Brubaker, Kyle E. C. Booth, Akihiko Arakawa, Fabian Furrer, Jayeeta Ghosh, Tsutomu Sato, Helmut G. Katzgraber

**Affiliations:** 1 Amazon Advanced Solutions Lab, Seattle, WA 98170 USA; 2 Research Division, Chugai Pharmaceutical Co., Ltd., Yokohama, Kanagawa 244-8602 Japan; 3 AWS Professional Services, Seattle, WA 98170 USA

**Keywords:** Biophysics, Computational biology and bioinformatics, Drug discovery, Molecular biology

## Abstract

The peptide-protein docking problem is an important problem in structural biology that facilitates rational and efficient drug design. In this work, we explore modeling and solving this problem with the quantum-amenable quadratic unconstrained binary optimization (QUBO) formalism. Our work extends recent efforts by incorporating the objectives and constraints associated with peptide cyclization and peptide-protein docking in the two-particle model on a tetrahedral lattice. We propose a “resource efficient” QUBO encoding for this problem, and baseline its performance with a novel constraint programming (CP) approach. We implement an end-to-end framework that enables the evaluation of our methods on instances from the Protein Data Bank (PDB). Our results show that the QUBO approach, using a classical simulated annealing solver, is able to find feasible conformations for problems with up to 6 peptide residues and 34 target protein residues (PDB 3WNE, 5LSO), but has trouble scaling beyond this problem size. In contrast, the CP approach can solve the largest instance in our test set, containing 11 peptide residues and 49 target protein residues (PDB 2F58). We conclude that while QUBO can be used to successfully tackle this problem, its scaling limitations and the strong performance of the CP method suggest that it may not be the best choice.

## Introduction

Peptide drugs are composed of short sequences of amino acids and are an important therapeutic modality for clinical treatments. These drugs have the potential to acquire higher binding affinity and selectivity, and to exhibit wider therapeutic windows than small molecule drugs, although there are drawbacks related to pharmacokinetic properties such as membrane permeability and metabolic stability^1,2^. Recently, it was reported that some pharmacokinetic issues could be resolved by the cyclization of peptides and incorporation of nonproteinogenic amino acids^3–5^. Therefore, cyclic peptides are increasingly expected to be a promising therapeutic modality for targeting both extracellular molecules, and intracellular biomolecules by oral administration^6,7^.

Structure-based drug design (SBDD), where a drug is designed based on three-dimensional structures of drug candidates and their target, is now widely applied to small-molecule drug discovery research. Thanks to advances in structural biology and artificial intelligence (AI), three-dimensional models of various target proteins are readily available for SBDD^8–12^.

In contrast to small molecules, cyclic peptides contain more rotatable bonds and thus have more possible conformations. Furthermore, their conformations depend on the surrounding environment (e.g., water or lipid membrane) and the nature of their target^5^. Consequently, building practical three-dimensional models of cyclic peptide-target complexes is a challenging task, though there are several docking tools for cyclic peptides tackling this challenge^13–18^.

A promising research direction approximates the conformation prediction problem as a combinatorial search over a fixed lattice^19–21^. Early versions of this approach model the peptide as a series of particles (points) in space, where each peptide residue is represented by a single particle, and the particles are placed on the vertices of a two-dimensional lattice grid. Any pair of particles that are within a fixed distance of each other contribute interaction potential according to whether both members of the pair are considered hydrophobic or hydrophilic (e.g., the HP model)^22^. Some straightforward extensions of this formulation include: i) a two-particle representation for the peptide residues, with one particle representing the residue’s main-chain backbone and the other representing the same residue’s side chain, ii) the use of Miyazawa-Jernigan (MJ) potentials as the interaction potentials between residues, and iii) the use of three-dimensional lattices^23,24^. The task is then to identify the placement of these peptide particles such that the MJ potential is optimized (minimized) while all known constraints, e.g., that no two particles occupy the same lattice vertex, are satisfied. This placement task is a combinatorial search problem that has been shown to be NP-complete^21,25^, imposing a severe limitation on the size of problems that can be solved with a classical computer. A potential avenue to overcome these scaling issues in the near term is to use noisy intermediate scale quantum (NISQ) technology such as quantum annealing^26–29^, quantum variational algorithms^30,31^, or Gaussian Boson sampling^32^. Common to most of these approaches is the formulation of the problem as a quadratic unconstrained binary optimization (QUBO) problem.

Early quantum computing approaches to protein folding employed a spatial encoding for amino acid conformations on 2D grids using the HP model^33^, though this method proved challenging to scale^34^. As an alternative to the spatial encoding, turn encoding approaches were explored^27,35^, beginning with a six-amino acid peptide implementation, followed by the introduction of the “turn ancilla” approach^26^. This methodology saw further development including an implementation for a 10-residue protein on a cubic lattice^34^, a sparse representation approach for QAOA on Rigetti Aspen^36,37^, an extension to two-particle coarse-grained residues on tetrahedral lattices^30^, and the introduction of a relative turn encoding with a qutrit mapping^31^.

Protein-peptide docking applications on quantum computers remain limited, with initial progress considering chaperone molecules’ impact on peptide conformations^27^, though this approach wasn’t generalizable to arbitrary protein-peptide complexes. We provide an expanded summary of the relevant literature in Supplementary A, including the summary Table S1.

In this work, we extend the lattice-based conformation search from the literature to incorporate the objectives and constraints associated with peptide cyclization and peptide docking with a target protein. We explore a number of formulations, including the “turn-ancilla” (Supplementary G) and “spatial” (Supplementary F) encodings, and based on preliminary model scaling analysis (described briefly in Supplementary E, with results in Figure S1), elect to extend the “resource efficient” turn encoding approach^30^. To compare the QUBO with a classical optimization approach, we also derive a constraint programming (CP) model for the problem. We implement an end-to-end framework that allows us to automatically process a problem instance from the Protein Data Bank (PDB) and produce conformation solutions with our QUBO-based and CP approaches^38^. We conduct an empirical assessment on a set of peptide-protein docked instances from the PDB and conclude that while the QUBO-based approaches can be used to model and solve the problem, they may not be the best-suited formalism. In contrast, the CP model is able to find the optimal solutions for all considered problem instances.

## Methods

### Problem definition

**Fig. 1:**
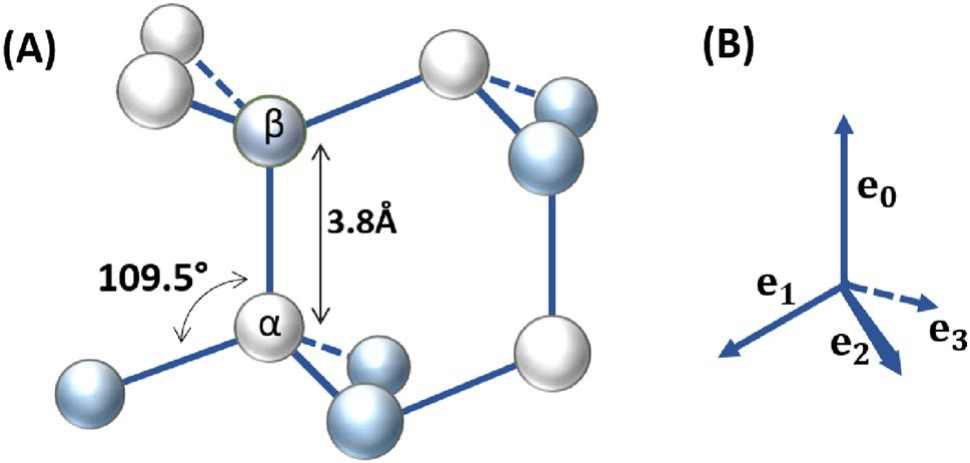
(**A**) Tetrahedral lattice structure $\mathcal {T}$ with vertices on sublattice $\mathcal {T}_\alpha$ indicated with white circles and $\mathcal {T}_\beta$ in blue circles. (**B**) Turn directions of the tetrahedral lattice.

We employ a two-particle coarse grain (CG) residue representation, describing main and side-chain particles for all residues except for a specified set of small residues (e.g., Glycine), for which we use a one-particle representation. We place these peptide particles on a three-dimensional tetrahedral lattice, for instance as depicted in Fig. 1, defined by the set of its vertices $\mathcal {T}$.

For a given problem instance, we consider a cyclic peptide consisting of an amino acid sequence $A = (A_1,\ldots ,A_N)$, where $A_i$ specifies the particular amino acid type for sequence location *i*, $A_i$ is adjacent to $A_{i+1}$, and *N* represents the number of amino acids. We use $A_i$ to refer to the main chain particle and use a superscript *s* to denote the corresponding side-chain particle $A^s_i$ that is assumed to be adjacent to $A_i$. Cyclization bonds of the peptide are expressed by pairs of residue numbers $C = \{(i_1,j_1),\ldots ,(i_K,j_K)\}$ for *K* specified pairs that are bonded, where typically $K=1$.

In this work, we consider a smaller, more focused area of the external protein, namely the “active site.” More details on how the active site is constructed for our experiments are provided in Supplementary H. This external protein active site is represented as $P=\{B_1,\ldots ,B_M\}$, where $B_i$ specifies the particular residue (i.e., amino acid type) at sequence location *i* and *M* is the number of residues.

We model the interaction energy between amino acid residues by Miyazawa-Jernigan (MJ) potentials^39,40^, which specify a fixed interaction potential between unique pairs of amino acid types. For simplicity of notation, we denote $\epsilon _{ij}$ to be the MJ interaction between residues $A_i$ and $A_j$. For the peptide, we restrict the interaction to non-bonded particles (i.e., main- and side-chain particles) that are first nearest neighbours (1-NN) on the lattice (i.e., a distance of one lattice edge); pairs of residues that are not 1-NN in the solution do not contribute any interaction energy.

Given the coordinates of the protein residues *P*, the lattice vertices $\mathcal {T_B} = \{t^B_{k}\}_k \subset \mathcal {T}$ that are “blocked” by the protein due to steric hindrance can be calculated using a specified blocking radius (e.g., 3.8Å). Similarly, using a specified interaction radius (e.g., 6.5Å), we can compute the lattice vertices $\mathcal {T_I} = \{t^I_{l}\}_{l} \subset \mathcal {T}$ on which the protein residues effect an interaction potential for the peptide. Here, we assume that all blocking points $\mathcal {T_B}$ are removed from $\mathcal {T_I}$, as the steric hindrance prohibits peptide residues from being placed on those vertices. We denote the total MJ interaction energy on peptide $A_i$ at the interaction site $t^I_l$ caused by the sum of the interactions between $A_i$ and the protein residues in $P_l$ by ${\tilde{\epsilon }}_{il}$.

An optimal solution to the optimization problem is a docking conformation for peptide *A* on tetrahedral lattice $\mathcal {T}$ in the presence of external protein *P* that minimizes the aggregate MJ interaction potential while satisfying the cyclization conditions *C* and ensuring peptide particles do not overlap with each other or with the set $\mathcal {T_B}$. A feasible conformation/solution is a conformation that satisfies all problem constraints but may have suboptimal MJ interaction potential. Additional details and assumptions of the problem definition can be found in Supplementary B.

### QUBO model

Various formulations of the problem without the external protein interaction (i.e., without docking) with different encoding strategies have been previously introduced and analyzed. We give a detailed overview of the different approaches in Supplementary A. Because of the beneficial scaling in the number of required variables, we start from the Hamiltonian derived in^30^ and introduce two additional terms, one for peptide cyclization, and another for the influence of the external target protein. We then use a locality reduction step to transform the higher-order terms into a quadratic form (Supplementary D). 

Our starting point is the Hamiltonian derived in^30^, which solves the peptide folding problem without cyclization and protein interaction:

1 $$\begin{aligned} H = H_\textrm{comb} + H_\textrm{back} \end{aligned}$$

where $H_\textrm{comb}$ combines the inter-peptide interaction energy with the no-overlap constraint penalty term for peptide particles that are more than three turns apart, and $H_{back}$ is the no-overlap constraint penalty term ensuring that subsequent main- and side-chain particles do not backtrack on each other. The Hamiltonian is based on a turn encoding of the peptide main- and side-chain particles, and the location of subsequent particles are defined by the turns on the tetrahedral lattice. For the explicit form of the Hamiltonian terms, we refer to Supplementary B.

As our first extension of the model presented in^30^, we add a cyclization constraint penalty term $H_\textrm{cycle}$ that enforces the cyclization bond $(i,j) \in C$, between given peptide residue pairs *i* and *j*. To do so, we define a distance function *d*(*X*, *Y*) that calculates the squared number of steps along the lattice between two lattice vertices *X* and *Y*, used to calculate distance between particles on the lattice. The distance is expressed in terms of the turn directions and the definition can be found in Supplementary B. We enforce cyclization by constraining the particles of the residue *i* and *j* to be 1-NN on the lattice (i.e., $d(A_i,A_j)=1$). For this we construct the term

2 $$\begin{aligned} H_\textrm{cycle} = \lambda _\textrm{cycle} (d(A_i,A_j) - 1)^p , \end{aligned}$$

where $\lambda _\textrm{cycle}$ is a strictly positive scalar used to regulate the penalty strength and *p* can be set to 1 or 2 depending on the solver properties. $H_\textrm{cycle}$ is equal to zero if the particles are one lattice step apart and adds a positive contribution otherwise as the non-overlap constraint forbids a distance zero.

For the peptide-protein interaction, we add MJ interaction energies for peptide residues residing on lattice points within the interaction range of the protein $\mathcal {T_I}$, and a constraint penalty term to represent steric hindrance on the sites $\mathcal {T_B}$ blocked by the protein. We leverage knowledge of the problem geometry and assume that for any point in $\mathcal {T_B}$ all 1-NN points not in $\mathcal {T_B}$ are in $\mathcal {T_I}$. For this to hold it is sufficient to choose blocking and interaction radii that differ by at least one lattice step. Necessarily, the peptide starts on a vertex outside of $\mathcal {T_B}$, and by our assumption, for any potential overlap with a point in $\mathcal {T_B}$ there must be an overlap with a point in $\mathcal {T_I}$.

Borrowing from the logic in $H_\textrm{comb}$, we design a Hamiltonian that adds a protein-peptide MJ interaction energy when a peptide particle resides on a point in $\mathcal {T_I}$ and none of its bonded 1-NN main or side-chain residues are on a blocking point in $\mathcal {T_B}$, and is strictly positive otherwise. If any bonded 1-NN particles do land on a point in $\mathcal {T_B}$, they will incur a positive penalty to the system energy, driving the optimizer to turn off the particle contributions which then zeroes out the MJ interaction. Additionally, we make sure that each interaction site can contribute at most one protein-peptide MJ interaction to the system, penalizing the placement of multiple peptide particles on the same interaction site.

We introduce an interaction variable $\eta _{il}$ ($\eta _{i^sl}$) for any valid combination of main (or side) chain particle *i* ($i^s$) and interaction site $t_l^{I} \in \mathcal {T_I}$, which acts as a switch in the minimization, turning on or off the energy contribution depending on if it is beneficial (negative contribution) or harmful (positive contribution) to the system. By valid combination, we mean that the site $t_l^{I}$ has sufficient non-blocked 1-NN sites required by the bonds of the peptide particle. We then define the peptide-protein Hamiltonian by

3 $$\begin{aligned} H_\textrm{protein}= & \sum _{i,l} \eta _{il} \Big (\epsilon _{il} (1 - \mu _1 d(A_i,t_l^{I})) + \mu _2 \sum _{X\in N(A_i)}\sum _{Y\in M_X(t_l^I)} \big (2 - d(X,Y)\big ) \Big ) \end{aligned}$$

4 $$\begin{aligned} & + \sum _{i,l} \eta _{i^sl} \Big (\epsilon _{il} (1 - \mu _1 d(A_{i^s}^s,t_l^{I})) + \mu _2 \sum _{Y\in M_{A_i}(t_l^I)} \big (2 - d(A_i,Y)\big ) \Big ) \end{aligned}$$

5 $$\begin{aligned} & + \mu _3 \sum _l \Big ( \sum _{i,j; i > j} (\eta _{il} \eta _{jl} + \eta _{i^sl} \eta _{j^sl}) + \sum _{i \ge j} \eta _{i^sl} \eta _{jl} \Big ). \end{aligned}$$

The outer sums run over all valid pairs of peptide main chain particle *i* and lattice points $t_l^{I} \in \mathcal {T_I}$, indexed by *l*. $M_X(t^I_l)$ denotes the set of all lattice sites 1-NN of $t_l^I$ that are either in $\mathcal {T_B}$ or do not have sufficient non-blocked 1-NN for the peptide particle *X* to be placed on them. In the second sum on line (4), the sum over all 1-NN of a side chain $A_{i}^s$ can be simplified as the only bonded particle is the corresponding main chain residue. The sums on line (5) are meant over all triples *l*, *i*, *j* such that the corresponding pairs in the summands are valid, and thus for which interaction variables exist.

The different terms in $H_\textrm{protein}$ and a proof that the Hamiltonian satisfies the requirements discussed above can be found in Supplementary C. Therein, we also derive the sufficient bounds for the penalty parameters $\mu _2 > -\epsilon _{il}/2$ for all *i*, *l* and

6 $$\begin{aligned} \mu _1 \ge 1 + 9 \mu _2 |N(A_i)||N(t_l^{I})|/|\epsilon _{il}|, \end{aligned}$$

where $|\cdot |$ applied to sets denotes the cardinality. Combining the Hamiltonian in Eq. (1) with the cyclization and protein interaction contributions, we arrive at our final Hamiltonian

7 $$\begin{aligned} H = H_\textrm{comb} + H_{back} + H_\textrm{cycle} + H_\textrm{protein} . \end{aligned}$$

### CP model

For each residue $A_i$ we associate a vector of integer variables $(x_i, y_i, z_i) \in \mathbb {Z}^3$ to describe the position of the residue on the lattice. We use *s* superscripts to identify side-chain elements where necessary (e.g., $x_i^s$ is the x-coordinate of the side-chain element for residue $A_i$).

We also introduce integer variables that represent the pairwise dimension distance between residues: $(x^{\delta }_{ij}, y^{\delta }_{ij}, z^{\delta }_{ij}) \in \mathbb {Z}^3, \forall i \ne j$. We introduce a set of boolean variables that identify if a pair of residues (or a residue and an external protein residue) is within an interaction threshold $\psi$ on the lattice, and thus have a contribution to the objective function: $\alpha _{ij} \in \{0,1\}, \forall i \ne j$. Finally, we use a set of boolean variables that flag if a pair of residues have a different location along the axes: $(x^{\ne }_{i,j}, y^{\ne }_{i,j}, z^{\ne }_{i,j}) \in \{0,1\}^3, \forall i \ne j$.

In contrast to linear programming-based model-and-solve technologies, CP supports logical constraints and non-linear relationships. The core objective, constraints, and variables of our CP model with a standard energy-minimizing objective function are expressed by:

8 $$\begin{aligned} \min \quad&\sum _{i<j: j \ge i+2 \in A \times A} \alpha _{ij}\epsilon _{ij} + \sum _{i, j^s \in A \times A } \alpha _{i,j^s}\epsilon _{i,j^s} \nonumber \\&+ \sum _{i^s, j^s \in A\times A} \alpha _{i^s,j^s}\epsilon _{i^s, j^s} + \sum _{i \in A, j \in P} \alpha _{i,j} \epsilon _{i,j} + \sum _{i^s \in A, j \in P} \alpha _{i^s,j} \epsilon _{i^s,j}&\end{aligned}$$

9 $$\begin{aligned} \text {s.t.} \quad\textsc {Table}\,\big((x_i, y_i, z_i), \mathcal {T} \setminus \mathcal {T_B}\big)\quad\forall i, i^s \in A \end{aligned}$$

10 $$\begin{aligned}&x^{\delta }_{ij} = |x_i - x_j|, y^{\delta }_{ij} = |y_i - y_j|, z^{\delta }_{ij} = |z_i - z_j|&\forall i \ne j \in A \times A \end{aligned}$$

11 $$\begin{aligned}&x^{\delta }_{i,i+1} + y^{\delta }_{i,i+1} + z^{\delta }_{i,i+1} = \Omega&\forall i \in A \setminus {A_N} \end{aligned}$$

12 $$\begin{aligned}&x^{\delta }_{i,i^s} + y^{\delta }_{i,i^s} + z^{\delta }_{i,i^s} = \Omega&\forall i \in A \end{aligned}$$

13 $$\begin{aligned}&x^{\delta }_{i,j} + y^{\delta }_{i,j} + z^{\delta }_{i,j} = \Omega&\forall (i,j) \in C&\end{aligned}$$

14 $$\begin{aligned}&x^{\ne }_{ij} \rightarrow x_i \ne x_j, y^{\ne }_{ij} \rightarrow y_i \ne y_j, z^{\ne }_{ij} \rightarrow z_i \ne z_j&\forall i\ne j \in A \times A \end{aligned}$$

15 $$\begin{aligned}&x^{\ne }_{ij} \vee y^{\ne }_{ij} \vee z^{\ne }_{ij}&\forall i \ne j \in A\times A \end{aligned}$$

16 $$\begin{aligned}&\alpha _{i,j} \rightarrow x^{\delta }_{i,j}, y^{\delta }_{i,j}, z^{\delta }_{i,j} \le \psi&\forall i \ne j \in A \times A \end{aligned}$$

17 $$\begin{aligned}&\alpha _{i,j} \rightarrow x^{\delta }_{i,j} + y^{\delta }_{i,j} + z^{\delta }_{i,j} \le \psi _{total}&\forall i \ne j \in A \times A \end{aligned}$$

18 $$\begin{aligned}&\alpha _{i,j} \rightarrow (x_i, y_i, z_i) \in \mathcal {I}_j&\forall i \in A, j \in P \end{aligned}$$

19 $$\begin{aligned}&(x_i, y_i, z_i) \in \mathbb {Z}^3_{+}&\forall i, i^s \in A \end{aligned}$$

20 $$\begin{aligned}&(x^{\delta }_{ij}, y^{\delta }_{ij}, z^{\delta }_{ij}) \in \mathbb {Z}^{3}_{+}&\forall i \ne j \in A \times A \end{aligned}$$

21 $$\begin{aligned}&(x^{\ne }_{ij}, y^{\ne }_{ij}, z^{\ne }_{ij}) \in \{0,1\}^3&\forall i \ne j \in A \times A \end{aligned}$$

22 $$\begin{aligned}&\alpha _{ij} \in \{0,1\}&\forall i \ne j \in A \times A \end{aligned}$$

Objective (8) expresses our core model objective function, which is to minimize MJ interaction energies of adjacent particles (since these interactions have negative values, minimization is desirable). This includes components for main-main, main-side, side-side, and external protein interactions. Constraint (9) uses the $\textsc {Table}$ constraint to ensure that the coordinates of each residue are placed on the points defined by the lattice but not affected by external protein steric hindrance, $\mathcal {T} \setminus \mathcal {T_B}$. The $\textsc {Table}$ constraint takes a tuple and maps it to a set of tuples (allowed value assignments). For example, if an external protein residue was located at coordinate (1, 1, 1), we could exclude this coordinate from $\mathcal {T}$ to ensure the solver does not place a residue there.

Constraint (10) links the pairwise distance variables to the location variables. Constraints (11)–(12) link the summation of the pairwise distance variables (for both main chain and side-chain) in each dimension to be equal to $\Omega$, the rectilinear distance of adjacent neighbors in the lattice. Similarly, Constraint (13) enforces neighboring rectilinear distance on residues included in cyclization bonds (for simplicity we omit some constraints for side-chain residues, but these are handled in similar fashion). Constraint (14) links the no-overlap variables and location variables, and Constraint (15) enforces the various non-overlap conditions for main-main, side-side, and main-side (namely that at least one coordinate value must be different between pairs). Constraints (16)–(17) link interaction variables, $\alpha$, to pairwise distance variables and bound their domains according to the interaction threshold. Constraint (18) enforces that a peptide residue must be located at one of the lattice vertices under the influence of the external protein (via projection), $\mathcal {I}_j$, for it to be considered interacting for the purposes of the objective function. Finally, Constraints (19) through (22) define the model variables and their domains.

### Problem instance construction

To measure the effectiveness of our approach, we take entries from the RCSB Protein Databank (PDB) and attempt to predict the peptide conformation found in each. By using PDB contents, we have ground truth solutions we can measure our predictions against. We chose a set of PDB instances that are short in length and have known cyclization specifications. A list of the PDB instances we consider in this work is given in Table 1.

**Table 1:** RCSB Protein Databank (https://www.rcsb.org/) peptide–protein complexes used for our experimental analysis. Cyclization for peptide 5LSO is specified between side-chain particles, and the cyclization of remaining instances all involve main chain particles.

PDB ID	Peptide chain	Cyclization	|*A*|	$|A^s|$	|*P*|
3WNE	P-K-I-D-N-G	P1-G6	6	5	26
5LSO	K-S-R-W-D-E	K1-E6	6	6	34
3AV9	S-A-K-I-D-N-L-D	S1-D8	8	8	28
3AVI	S-L-K-I-D-N-M-D	S1-D8	8	8	32
3AVN	S-H-K-I-D-N-L-D	S1-D8	8	8	28
2F58	H-I-G-P-G-R-A-F-G-G-G	H1-G10	11	6	49

Instances in the PDB repository describe cyclic protein complexes using atomic-level coordinates in a standardized format. Before the PDB contents can be incorporated into the problem, they must be processed and projected onto the lattice. We provide a summary of the pipeline constructed to achieve this, but further details can be found in Supplementary H. The first step is to separate the information in the PDB file into two components: i) the peptide, and ii) the target protein (i.e., the protein that the peptide docks onto), as shown in Figure S2a. The next step is to identify the protein active site (Figure S2b), which constitutes a smaller, more focused area that the peptide is likely to use for docking.

Each atom in the peptide or protein active site is then classified as belonging to main chain or side chain, and the weighted centroid of each grouping is calculated, yielding a 2-particle representation for each amino acid in each structure (Figure S2c). The last step is to produce the tetrahedral lattice that discretizes the search space for our optimization methods (Figure S2d). Our approach for placing the tetrahedral lattice is described in Algorithm S1. The lattice is then filtered according to the current active site coordinates *P* and steric clash radius to remove vertices that would clash with the target site $\mathcal {T_B}$, yielding the final lattice structure. The problem instance can then be characterized by the peptide main and side chains, filtered tetrahedral lattice coordinates $\mathcal {T}'$ (with origin $t_1$), and shifted protein active site coordinates, *P*.

### Solver implementation details

To solve the QUBO model, we first use a reduction step to transform the (typically) high order polynomial Hamiltonian into a quadratic form, which can introduce overhead in the form of ancilla variables. To keep problem size tractable, we additionally employ an energy impact decomposition strategy to decompose the QUBO into smaller sub-problems (sub-QUBOs), such that it could potentially be solved using current quantum hardware. Then we submit each sub-problem to a simulated annealing solver to sample solutions and return the best solution found. The results from each sub-problem are injected back into the global solution to generate an updated solution state, and the global solution is passed to a local Tabu search for further refinement. This combined Simulated Annealing and Tabu search process is repeated until termination criteria are met (e.g., some number of global steps is reached). These steps are implemented by leveraging the D-Wave hybrid python library, including the EnergyImpactDecomposer, the SimulatedAnnealingSampler, and the TabuProblemSampler.

As part of our experimentation, we ran hyperparameter optimization (HPO) for the QUBO model over the Hamiltonian *H* terms $\lambda _\textrm{cycle}$, $\lambda _\textrm{back}$, $\mu _{2}$, $\mu _{3}$, *p*, the QUBO decomposition size $sub\_qubo\_size$, and the simulated annealing solver parameters $num\_reads$, $num\_sweeps$. This HPO process sought to minimize a custom loss function, which was the sum of the Hamiltonian energy *H* of the best solution found and a scaled (1000x) count of violations observed in the solution. See Supplementary D, and Supplementary H for more details on each step outlined here.

To solve the CP model we use the Google OR-Tools CP-SAT solver^41^ with default settings. Logical ‘implies’ (e.g., $\rightarrow$) is implemented using the OnlyEnforceIf method, and the logical disjunctions in Eq. (15) are implemented as BoolOR constraints.

## Experiments

We conduct an experimental analysis on the peptide instances summarized in Table 1 using the instance generation pipeline and models discussed in “Methods”. We include results and analysis for instances that both exclude and include the external protein, only the latter of which can be considered peptide docking.

We assess feasible conformations produced by the approaches in terms of both their MJ interaction value and the root mean square distance (RMSD) compared against the coordinates of the real peptide (sourced from the PDB file) in Euclidean space. RMSD measures the average distance between the coarse-grained particles of the real (PDB) peptide and the peptide produced by our approach. The equation for RMSD is defined as follows:

23 $$\begin{aligned} \textrm{RMSD}(\textbf{v}, \textbf{w}) = \sqrt{\frac{1}{n}\sum _{i=1}^{n} ||v_i - w_i||^2}, \end{aligned}$$

where $\textbf{v}$ denotes the coordinates for the real peptide and $\textbf{w}$ denotes the coordinates of the predicted peptide, and the RMSD represents the $\ell ^2$-norm of the difference between both vectors.

It is important to note that while our approach optimizes for aggregate MJ energy, the practical success of a conformation is typically gauged using RMSD or other molecular similarity metrics.

### Excluding the external protein

By removing the external protein from the problem (and the associated penalty term $H_{protein}$), we reduce the QUBO model size significantly, thereby making it easier to solve. For instance, for peptide 3WNE, the number of variables in the QUBO formulation without the external protein is 152, and the number of variables when the external protein is included is 596.

As our resource-efficient turn encoding approach is an *unconstrained* formulation that leverages penalty terms in the objective, it is possible for the solver to return states corresponding to peptide conformations that violate problem constraints. As the problem size (number of variables) grows, it becomes harder for the solver to find high-quality states corresponding to feasible conformations. When the external protein is excluded, the QUBO solver is able to find the optimal solution in at least one shot for each problem instance.

Table 2 summarizes the solver’s ability to find feasible conformations, and also reports on the best conformation MJ energy found per problem instance. While we did run HPO over these problems, we found that it was possible to use one general set of hyperparameters and yield feasible results.

**Table 2:** QUBO results with no external protein present. The table shows the fraction of states corresponding to feasible conformations and the lowest MJ interaction across all 100 HPO jobs, with 10 shots per job, for each problem instance.

PDB ID	Feasible fraction	Best MJ interactions
3WNE	1.0	$-1.87$
5LSO	1.0	$-5.97$
3AV9	0.55	$-6.64$
3AVI	0.61	$-8.54$
3AVN	0.55	$-6.88$
2F58	0.08	$-6.02$

Table 3 provides a summary of feasible conformation quality produced by the approaches in terms of MJ interaction and RMSD values; QUBO solver shots that resulted in constraint violations are not included. To provide context for these results, we also include the optimal MJ and associated RMSD attained by our CP-based approach, where the CP model is optimizing aggregate MJ interaction energy, as in Objective (8), with the first residue anchored to the origin to facilitate fair comparison with the QUBO results.

Removing the external protein removes the external (protein) MJ interaction potentials associated with the “correct” conformation; meaning there is no force driving the solvers to a conformation that is spatially aligned with the ground truth PDB result. This also means that the peptide could fold into a stable, feasible conformation in a number of directions that it otherwise would be driven away from (in the presence of the external protein). Removal of the protein also removes the protein blocking influence over the peptide, meaning a feasible state in this context could actually be infeasible if the external protein were considered.

**Table 3:** No protein MJ interaction and RMSD values for QUBO approach vs. CP approach. The CP model is anchored to the same starting point as QUBO method to enable fair comparison. The CP results include runs with interaction radius limits set to one grid step (CP 1-NN) and the default interaction radius of 6.5Å (CP). $t_\textrm{QUBO}$ is the time used to solve a given problem instance (excluding HPO). For all results, we report the mean and standard error of each metric across 10 shots, using a fixed set of hyperparameters for the QUBO model across all PDBs. The number of feasible results per PDB are 3WNE = 10, 5LSO = 10, 3AV9 = 4, 3AVI = 6, 3AVN = 8, 2F58 = 2.

	MJ interactions			RMSD (Å)			
PDB ID	QUBO	CP (1-NN)	CP	QUBO	CP (1-NN)	CP	${t_\text {QUBO}}$ (s)
3WNE	$-1.87\pm 0.00$	$-1.87\pm 0.00$	$-46.30\pm 0.00$	$10.03\pm 0.20$	$7.77\pm 0.10$	$7.51\pm 0.00$	$126\pm 7$
5LSO	$-5.93\pm 0.04$	$-5.97\pm 0.00$	$-53.56\pm 0.00$	$13.31\pm 0.16$	$13.20\pm 0.13$	$13.60\pm 0.06$	$161\pm 11$
3AV9	$-6.18\pm 0.40$	$-6.64\pm 0.00$	$-101.09\pm 0.00$	$11.36\pm 0.16$	$9.92\pm 0.27$	$9.56\pm 0.20$	$156\pm 16$
3AVI	$-7.45\pm 0.64$	$-8.54\pm 0.00$	$-114.71\pm 0.00$	$9.65\pm 0.52$	$9.39\pm 0.04$	$8.87\pm 0.00$	$191\pm 18$
3AVN	$-6.52\pm 0.21$	$-6.88\pm 0.00$	$-102.01\pm 0.00$	$10.60\pm 0.45$	$10.75\pm 0.26$	$12.28\pm 0.16$	$325\pm 15$
2F58	$-6.18\pm 0.11$	$-13.61\pm 0.00$	$-134.19\pm 0.00$	$11.52\pm 0.33$	$11.39\pm 0.38$	$10.83\pm 0.30$	$237\pm 23$

We note an obvious gap between the QUBO and CP MJ energies using the default interaction radius of 6.5Å. When the CP model uses a reduced interaction radius (one grid step), we see similar performance between the QUBO and CP (1-NN) results, showing that the QUBO model is able to effectively solve the problems, though it is slightly worse on average. We note that 2F58 does not follow this trend, as the CP results are markedly better than the QUBO results. We also see that the QUBO runtimes are fairly consistent across peptides, with 3WNE being the fastest at 126 s ($\sim$2 min), and 3AVN being the slowest at 325 s ($\sim$5.5 min). This leads to the expectation that the solver can solve problems without the external protein within $\sim$5 min (not including HPO time).

### Including the external protein (docking)

Here we conduct docking experiments where the external protein is included. The QUBO solver was only able to find states corresponding to feasible conformations for some problem instances, so we first report on the number and type of constraint violations observed. For each problem instance, we determine the shot that resulted in the most optimized QUBO objective function value and then assess the breakdown of constraint violations for that state. We report violations for the number of overlaps involving pairs of peptide residues (# Overlaps), the number of steric clashes between a peptide residue and the external protein (# Protein Clash) and whether the cyclization constraint is satisfied by the state.

As shown in Table 4, the QUBO approach yields states associated with feasible conformations for the smaller peptides 3WNE and 5LSO, but struggles as problem size increases. For instance, the best MJ state for 3AV9 has six overlaps of the peptide, two steric clashes with the external protein, and the cyclization was not satisfied. Because 3AV9 involves eight main chain residues and eight side chain residues (for a total of sixteen peptide residues), there are ${\tiny {16 \atopwithdelims ()2}} = 120$ possible pairs of overlapping residues. This means that the state returned by the QUBO solver satisfies 114/120 $(95.0\%)$ of the peptide overlap constraints. When considering the external protein, the 3AV9 instance involves 28 external protein residues indicating a possible $16 \times 28 = 448$ steric clash combinations. Of these, the state violates 2, satisfying over 99.5% of these constraints. Note that this calculation provides a lower bound, and is useful for illustrative purposes; the projection of blocking influence onto the lattice often yields more blocking sites on the lattice than the number of residues present, and the peptide could potentially clash with any of those.

**Table 4:** With protein QUBO state constraint violations by instance. For each peptide, the shot associated with the best MJ interaction is selected (out of 10 shots total). Overlaps and protein clash calculated for all relevant pairs $(i <j)$. % Satisfied (% Satis.) calculated as the number of conflicts observed divided by the total number of possible conflicts. Peptide residue count (# Peptide) includes main and side chain residues.

PDB ID	# Overlaps (% sat.)	# Protein clash (% sat.)	Cyclization
3WNE	0 (100.0%)	0 (100.0%)	True
5LSO	0 (100.0%)	0 (100.0%)	True
3AV9	6 (95.0%)	2 (99.6%)	False
3AVI	9 (92.5%)	1 (99.8%)	True
3AVN	5 (95.8%)	1 (99.8%)	False
2F58	14 (89.7%)	2 (99.8%)	True

A summary of feasible conformation quality with the target protein included is given in Table 5. First, we note that the QUBO model yields less favorable MJ energies than the equivalent CP (1-NN) model, and is expectantly worse than the default CP model. Second, the QUBO model produced results that were closer than the CP (1-NN) model to the PDB solution per the RMSD metric for 5LSO, and comparable RMSD for 3WNE.

**Table 5:** Aggregated mean and standard error of MJ interaction and RMSD values for QUBO approach vs. CP approach, across 10 shots. The CP model is anchored to the same starting point as QUBO method to enable fair comparison. CP results are shown for nearest neighbor interaction (CP 1-NN) similar to the QUBO model, and a distance based interaction radius of 6.5Å (CP). $t_\textrm{QUBO}$ is the time used to solve a given problem instance (excluding HPO). For the QUBO model, the same hyperparameters are used for all shots within a PDB, and we report the mean and standard error of the feasible shots for 3WNE and 5LSO; the QUBO model found no results (feasible states) for the remaining PDBs. The number of feasible results per PDB are 3WNE = 3, 5LSO = 2.

	MJ Interactions			RMSD (Å)			
PDB ID	QUBO	CP (1-NN)	CP	QUBO	CP (1-NN)	CP	$t_\text {QUBO}$(s)
3WNE	$-28.53\pm 5.02$	$-42.64\pm 0.00$	$-76.86\pm 0.00$	$8.88\pm 0.36$	$8.48\pm 0.00$	$8.48\pm 0.00$	$268\pm 18$
5LSO	$-17.93\pm 0.00$	$-23.26\pm 0.00$	$-62.41\pm 0.00$	$7.68\pm 0.00$	$10.29\pm 0.00$	$10.29\pm 0.00$	$213\pm 8$
3AV9	–	$-40.44\pm 0.00$	$-133.74\pm 0.00$	–	$9.89\pm 0.02$	$8.98\pm 0.00$	$642\pm 36$
3AVI	–	$-62.34\pm 0.00$	$-159.81\pm 0.00$	–	$9.90\pm 0.02$	$8.74\pm 0.01$	$712\pm 39$
3AVN	–	$-55.12\pm 0.00$	$-136.83\pm 0.00$	–	$8.44\pm 0.01$	$8.34\pm 0.03$	$672\pm 36$
2F58	–	$-4.02\pm 0.00$	$-215.91\pm 0.00$	–	$12.08\pm 0.01$	$10.42\pm 0.00$	$1595\pm 146$

**Fig. 2:**
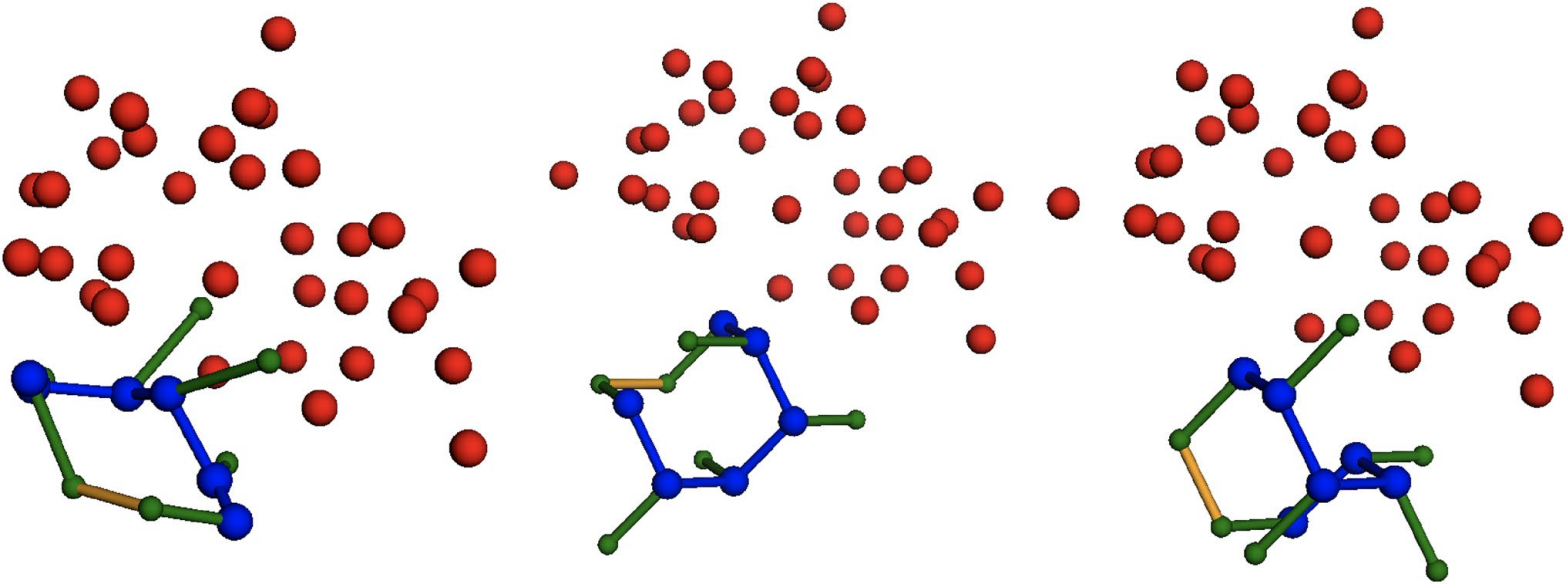
5LSO result visualization. Comparing the true PDB peptide (left) against the QUBO model result (middle) and the CP-MJ 1-NN result (right). The peptide is represented with blue and green dots (main and side-chain, respectively), and the red dots are the external protein residues.

This may seem surprising, but we must consider that the CP results shown here are for runs of the CP model where the first peptide residue is *anchored* to the lattice origin. Recall that the anchor was introduced in order to provide a more direct and fair comparison between QUBO and CP models. Because of this anchoring, while working to maximize interactions with the external protein, the CP models create high-quality solutions that are rotated off of alignment with the ground truth PDB result. An example of this is shown in Fig. 2, with additional examples in Supplementary Figures S3, S4, S5, S6, S7,. Finally, we note some significant variance in solver runtimes in this case, with 5LSO being the fastest at 213 s ($\sim$4 min), and 2F58 being the slowest at 1595 s ($\sim$27 min).

Our results demonstrate that mapping protein optimization type problems to QUBOs inherently makes these harder to solve, not only because penalty terms are hard to construct and parameters hard to tune, but also because any solver must find sufficiently high-quality (e.g., low energy) states to avoid the violation of hard constraints, which is not always possible, especially for such large systems. This also indicates that protein-related problems are not well suited for QUBO-based solvers, e.g., quantum annealers^42^.

## Discussion

In this work, we have explored solving the cyclic peptide docking problem on a fixed lattice in a general manner using a QUBO approach, and in parallel constructed a novel constraint programming (CP) formulation. To do this, we have built on previous work^30^ to include a cyclization constraint term and a general peptide-protein interaction term, which had not been done before. We find that wrapping the combined distance checks on interaction and blocking sites with an ancilla bit (to turn on/off), as described in “Methods”, is the most efficient approach to include the protein interactions.

The PDB instances used in this work, outlined in Table 1, were chosen due to their relatively short length and known cyclization specification. By choosing short length instances, we kept the problem size small to effectively evaluate the model’s properties and ability to yield feasible states as we added complexity through the external protein interactions. We note that this work included a relatively small sample set that adhered to these requirements, and so an important next step is to expand the set of PDB instances to, for example, 1NPO, 2CK0, 3VVS, and 4K8Y. Further, we note that the inclusion of nonproteinogenic amino acids should be straightforward with respect to the pipeline logic, because all existing pipeline steps would apply equally to these amino acids, with the exception of any potentially odd-shaped amino acids that might require bespoke particle centroid calculations. However, inclusion of any nonproteinogenic amino acids would require the use of some other inter-particle attraction measure, as MJ values do not exist for such amino acids. This would require further consideration before any peptides containing such amino acids could be solved for.

One of the primary difficulties of modeling cyclic peptide docking problem in the QUBO formalism is the construction of disjunctive expressions that turn ‘on’ or ‘off’ various components of the function. The problem requires inclusion or exclusion of forces (energies) according to distance thresholds (e.g., overlap penalties when $d_{ij}=0$ or pairwise MJ interactions when $d_{ij}=1$), which exist at single points in space and are zero otherwise. These would be effectively modeled by a (set of) delta function(s). Unfortunately, there is no easy or efficient way to include these types of forces naturally in a Hamiltonian expression. Instead, we must add ancilla bits which leads to additional overhead in the problem representation and makes it harder to solve.

An important simplification to the cyclic peptide docking problem is the restriction of placement of the residues on vertices of a lattice structure. In practice, peptides are not constrained to a particular rigid underlying structure, they simply abide by steric forces preventing the molecules from clashing with one another. Therefore, the introduction of any lattice structure introduces some error between the model result and the true PDB solution. This is one driver of the large RMSD values seen in Tables 3 and 5.

When the external protein is removed, all peptides tested can be solved with the QUBO representation. As shown in Table 3, the QUBO solver was able to find the optimal MJ potential in five out of six cases, with 2F58 being the exception with a total MJ potential of $-6.02$ compared to $-13.61$ from the CP model (approx. 55.77% gap). We also note similar RMSD values between the QUBO and CP (1-NN) models, with QUBO performing best per RMSD on 3AVI at 9.65Å, and CP performing best on 3WNE at 7.77Å.

When the external protein is included, the QUBO approach finds feasibility for only two out of five peptides (3WNE, 5LSO). Within these, as Table 5 shows, the MJ potentials were both worse than the CP (1-NN) solutions, with a gap of approximately 33.10% for 3WNE and approximately 16.52% for 5LSO. The RMSD values for QUBO results were comparable to those of CP (1-NN) on 3WNE, and better than CP (both models) on 5LSO. This suggests that MJ-optimized solutions may not always perform well with respect to RMSD against the PDB ground truth, especially when anchored to what might be a suboptimal starting location. Further, it is worth noting that in practice, an RMSD greater than 4Å is considered to be a non-match and not practically useful.

We note that it is not particularly surprising that the QUBO approach struggles to find feasible states for some of the peptides. As the peptide size increases, the size of the QUBO model increases super-linearly (Supplementary E), thus making it harder to solve. Because the simulated annealing solver is a local, sequential search algorithm, the time it needs to converge increases with problem size, and so with a fixed time budget, the probability of finding a feasible state decreases. This is evident in Table 2, showing the decreasing feasible fraction with increasing peptide size. Some strategies to increase the probability of finding a feasible state would be to increase the memory of the underlying compute node, and to increase the runtime allowance of the solver.

Overall, the inability of the QUBO model to consistently find states corresponding to feasible conformations in the presence of an external protein is a significant limitation of the approach, which also implies that quantum optimization techniques^42^ that leverage QUBO representations might not be the most efficient for this class of application. In contrast, the CP approach is scalable and has the potential to deliver new insights into peptide-protein interactions.

## Supplementary Information

Supplementary Information.

## Data Availability

We source all our raw data from the RCSB Protein Databank (PDB), which can be found at https://www.rcsb.org/. Due to IP concerns, we do not provide exports of our processed PDB intermediates, but we do provide a description of the steps necessary to recreate the data in Supplementary H.
