# Supplementary material for: Quadratic unconstrained binary optimization and constraint programming approaches for lattice-based cyclic peptide docking
Source: Sci Rep. 2025 Jul 1;15:20395. doi: 10.1038/s41598-025-05565-1 (PMC12214603; doi:10.1038/s41598-025-05565-1)
Supplement: Supplementary file 1 — Supplementary Information. [file 41598_2025_5565_MOESM1_ESM.pdf]

Supplemental Material: Quadratic unconstrained binary optimization and constraint programming approaches for lattice-based cyclic peptide docking

J. Kyle Brubaker[1], Kyle E. C. Booth[*,1], Akihiko Arakawa[2], Fabian Furrer[1], Jayeeta Ghosh[3], Tsutomu Sato[2], Helmut G. Katzgraber[1]

[1]*Amazon Advanced Solutions Lab, Seattle, Washington, 98170, USA*
[2]*Research Division, Chugai Pharmaceutical Co., Ltd.,Yokohama, Kanagawa, 244-8602, Japan*
[3]*AWS Professional Services, Seattle, Washington, 98170, USA*
[*]Corresponding author (Email address: **kybooth@amazon.com**)

A. Related work

Perdomo et al. [1] solved for the optimal conformation of an amino acid sequence on a two-dimensional grid, using the hydrophobic-polar (HP) interaction model and a one-particle representation leveraging quantum computing techniques. This work introduced the idea of a spatial encoding, wherein the coordinates on the grid are mapped to sets of bits, and the goal is to assign bits to each residue such that their placement on the grid minimizes the system energy (thereby maximizing the number of hydrophobic (H) residues that interact). While this spatial encoding might be the most intuitive encoding—it is straightforward to map a solution bitstring back to a series of x and y coordinates—it is not very scalable for larger peptides in three-dimensional lattice geometries because of the bit requirements to encode the position in each dimension [2].

An alternative to the spatial encoding is the turn encoding, which defines the peptide geometry by a series of successive turns connecting one particle with the next, or to its respective side chain particle, see Ref. [3]. In the turn encoding there are a fixed number of turn directions, according to the lattice geometry, and each turn direction is mapped to a set of bits. The solution bitstring defines the full series of turns the peptide particles take, starting from the first residue, to yield the final conformation. This encoding offers an improved variable scaling proportional to the peptide length [2] relative to the spatial encoding. Perdomo *et al.* successfully applied this turn encoding to a small peptide of six amino acids, formulated as a one-particle coarse grained (CG) representation on a square lattice using a quantum annealer developed by D-Wave Systems Inc. [4, 5].

Babbush *et al.* [3] provide an overview of multiple problem formulations based on turn encodings, and introducing an approach using slack variables to implement the overlap constraint as a penalty term, referred to as the "turn ancilla" approach. Babej *et al.* [2] applied the turn ancilla approach to the cubic lattice with a dense turn encoding and a one-particle CG representation to fold a 10-residue protein on a quantum annealer. Fingerhuth *et al.* [6] extended this approach, using a one-hot encoding for each turn direction, which yielded a sparser representation allowing for alternative calculations of the Hamiltonian components. This was important for formulating the problem for a quantum alternating operator ansatz (QAOA) approach, which could be run on the Rigetti Aspen quantum circuit device [6, 7]. Robert *et al.* [8] applied the turn encoding to model two-particle CG residues on a three-dimensional tetrahedral lattice, using a more variable efficient method to implement the overlap constraint than the turn ancilla approach. They then applied a Variational Quantum Eigensolver (VQE) algorithm on the IBM Q 20-qubit circuit-based quantum device to find minimal energy states. Due to the limited number of qubits available, they reduced the problem scale in their experiments to a peptide consisting of seven residues, using the one-particle CG representation. Boulebnane *et al.* [9] used a heavy atom representation of amino acids, wherein hydrogen atoms are grouped together with larger atoms such as carbon, oxygen, or nitrogen, placed on a tetrahedral geometry, modeling peptide interactions via the Lennard-Jones interaction potential. Furthermore, they introduced a relative turn encoding that maps the three possible directions into a qutrit and applied QAOA to solve the minimization problem. This was a more granular problem representation, and therefore a more realistic approach, but the scaling requirement was high, even for a small four-residue peptide.

There has been limited research in formulating lattice-based models for protein-peptide docking experiments that could be run on quantum computers. Perdomo *et al.* [4] introduced some related ideas on accounting for chaperone molecules that impact the resulting peptide conformations. This can be seen in their Supplemental Section I.C, where they assume the placement of the first two residues, and solve for the remaining length of four, accounting for a hypothetical molecule that blocks multiple points on the grid. By knowing their starting point, they can construct a set of equations that would penalize solutions that would lead into this region or would incur a backtrack. The work enumerates all such cases and constructs a composite formula to handle them. However, this is an intractable approach and is not applicable to arbitrary peptide and protein complexes.

Our work represents an extension of the problem formulation to the protein-peptide docking problem that applies to *arbitrary peptides and proteins*. For a concise overview of the related work cited we refer to Table J, which summarizes problem setup and contribution.

Reference	Year	Encoding	Lattice	CG model	Interaction
Perdomo-Ortiz *et al.* [1]	2008	Spatial encoding	2D Square	1-particle	HP<br>Intra.
Babbush *et al.* [3]	2012	Turn ancilla,<br>turn circuit,<br>diamond	2D Square	1-particle	HP<br>Intra.
Perdomo-Ortiz *et al.* [4]	2012	Turn ancilla	2D Square	1-particle	MJ<br>Intra.
Fingerhuth *et al.* [6]	2018	One hot<br>turn encoding<br>QAOA	2D Square	1-particle	HP and MJ<br>Intra.
Babej *et al.* [2]	2018	Turn ancilla,<br>also introduced<br>turn circuit and<br>spatial nested shell	3D Cubic	1-particle	MJ<br>Intra.
Robert *et al.* [8]	2021	Turn encoding<br>(sparse and dense)<br>QAOA	3D Tetrahedral	2-particle	MJ<br>Intra.
Irback *et al.* [10]	2022	Spatial encoding	2D Square	1-particle	HP<br>Intra.
Boulebnane *et al.* [9]	2022	Relative turn<br>encoding	2D Square and<br>3D Tetrahedral	heavy atomic	LJ<br>Intra.
**Our work:**<br>Cyclic peptide docking	2024	Turn encoding	3D Tetrahedral	2-particle	MJ<br>Intra.<br>+ Inter.

Table S1: Literature review of related work. Here "Intra." refers to intramolecular interactions and "Inter." refers to intermolecular interactions. 2D refers to a two-dimensional square lattice and 3D to a three-dimensional cubic one, respectively.

B. Resource-efficient turn encoding

Here we provide the building blocks of the resource efficient formulation by more thoroughly defining the elements of H_{comb}, as initially described in Ref. [8].

B.1. Turn encoding on a tetrahedral lattice

At each vertex there are four different turns that connect to the neighboring vertices (recall Figure 1). Because of the geometry of the tetrahedral lattice, the direction of these turns alternate with each step taken on the lattice. Therefore, we introduce alternating sub-lattices $\mathcal{T}_\alpha$ and $\mathcal{T}_\beta$ with counter-directional turn directions. On vertices of $\mathcal{T}_\alpha$, we chose an arbitrary but fixed enumeration of the four possible turns $(0, 1, 2, 3)$. We then observe that on vertices $\mathcal{T}_\beta$ the turn directions are inverted and label them so that turn $k \in (0, 1, 2, 3)$ on $\mathcal{T}_\beta$ is the inverse of k on $\mathcal{T}_\alpha$. Hence, if turn k points in the direction of vector e_k on $\mathcal{T}_\alpha$ then turn k on $\mathcal{T}_\beta$ points in the inverted direction $(-e_k)$. On vertices of $\mathcal{T}_\mathcal{A}$, we denote the four turn directions by $t \in (0, 1, 2, 3)$ and on vertices $\mathcal{T}_\mathcal{B}$ by $t \in (\bar{0}, \bar{1}, \bar{2}, \bar{3})$, where turn direction k and $\bar{k}$ are in opposite directions.

Because the tetrahedral lattice has four turn directions, they can be bijectively mapped to pairs of bits via the encoding $(0, 1, 2, 3) \mapsto (00, 01, 10, 11)$. The conformation of the main-chain particles of a peptide of length N is characterized by $N-1$ turns, and thus needs $2(N-1)$ bits to be encoded $(q_1q_2...q_{2N-3}q_{2N-2})$. Note that for the following formulas we assume that the main chain of the peptide starts on sub-lattice $\mathcal{T}_\alpha$ and the number of turns uniquely specifies the sub-lattice a particle is located on.

To encode the location of the side-chain particles, we use the same turn encoding applied to the turn s_i connecting main- and side-chain particles A_i and A_i^s. Hence, the side chain is represented by $2N^s$ bits $\tilde{q}_1\tilde{q}_2....\tilde{q}_{2N^s-1}\tilde{q}_{2N^s})$, where $N^s \leq N$ is the number of side chain particles.

In order to calculate MJ interactions and enforce constraints, we need to express the distance between any lattice vertices in terms of our turn encoding. We will again follow Ref. [8] and use turn indicators f_k, defined by $f_k(t) = 1$ if and only if t is a turn in direction k and otherwise 0. These functions can be easily expressed in terms of the bit values q_1, q_2 of the turn t by $f_0(t) = (1-q_1)(1-q_2)$, $f_1(t) = q_2(q_2 - q_1)$, $f_2(t) = q_1(q_1 - q_2)$ and $f_3(t) = q_1q_2$.

Fixing the lattice origin at the start of the peptide, any lattice vertex v can be identified by a four-dimensional turn vector $x = (x_0, x_1, x_2, x_3)$, where x_k denotes the number of required turns in direction k from the origin in order to reach v. We keep track of the inverted directions on different sub-lattices by setting $x_k = 1$ for a turn in direction k on sub-lattice $\mathcal{T}_\alpha$, and $x_k = -1$ for a turn in direction $\bar{k}$ on sub-lattice $\mathcal{T}_\beta$. For any A_i, we can express x_k using the turn indicators

$$x_k(A_i) = \sum_{l=1}^{i-1} (-1)^{l+1} f_k(t_l) \tag{S1}$$

and for a corresponding side chain by

$$x_k(A_i^s) = x_k(A_i) + (-1)^{i+1} f_k(s_i)\,. \tag{S2}$$

Using the turn vectors, we introduce the squared distance between any A_i and A_j by

$$d(A_i, A_j) = \sum_k \big(x_k(A_j) - x_k(A_i)\big)^2\,. \tag{S3}$$

Note that the squared distance is used to ensure the distance value is non-negative, but comes at the cost of introducing higher-order polynomial terms into the Hamiltonian, which leads to variable overhead in the reduction of the problem to a QUBO.

This distance function can also be applied to arbitrary lattice points v and w with turn vectors x and y as $d(v, w) = \sum_k (y_k - x_k)^2$. An important property of the squared distance, resulting from the geometry of the tetrahedral lattice, is that the distance between any pair of grid points that are two turns apart is equal to 2 and cannot be 4 because two consecutive steps in the same direction lead to 0 (recall $x_k = 1$ on $\mathcal{T}_\mathcal{A}$ and $x_k = -1$ on $\mathcal{T}_\mathcal{B}$).

B.2. Hamiltonian construction

The Hamiltonian describing the optimization problem contains two categories of terms: the actual energy terms to be minimized, and the constrained penalty terms enforcing the validity of the states. The energy terms account for the interaction energies of non-bonded peptide particles and the peptide-protein interaction energy. The constrained penalty terms are constructed such that they are equal to zero if the constraints are satisfied and strictly positive otherwise.

Our starting point is the Hamiltonian derived in Ref. [8], which solves the peptide folding problem without cyclization and peptide-protein interaction. The Hamiltonian is given by

$$H = H_{\text{comb}} + H_{\text{back}} \tag{S4}$$

where H_{comb} combines the inter-peptide interaction energy with the no-overlap constraint penalty term for peptide particles that are more than three turns apart, and H_{back} is the no-overlap constraint penalty term to avoid backtracking of subsequent main and side chain turns. For completeness, and because the formulas including side chains are not explicitly given in Ref. [8], we provide them here.

B.2.1. Backtracking constraint

We introduce the helper function $T(t, t') = \sum_{k=0}^{3} f_k(t) f_k(t')$ that is equal to 1 if turns t and t' are in the same direction and else 0. We can then formulate the no backtracking condition as

$$H_{\text{back}} = \lambda_{\text{back}} \Big(\sum_{i=1}^{N-2} T(t_i, t_{i+1}) + \sum_{i=1}^{N-1} T(t_i, s_{i+1}) + \sum_{i=1}^{N-1} T(t_i, s_i) \Big)\,, \tag{S5}$$

where the first sum adds a positive penalty for backtracking along main-chain turns, the second for backtracking of side-chain turns on the main-chain turns, and the third for side-chain turns in the same direction as main-chain turns. Here we assume that every main-chain particle has a side-chain particle, otherwise the terms containing s_i are omitted. The constraint penalty parameter $\lambda_{\text{back}} > 0$ can be adjusted and determines the Hamiltonian contribution of a constraint violation due to backtracking.

B.2.2. Inter-peptide interaction energy combined with no-overlap constraint

The inter-peptide interaction energies are added for all non-bonded particle pairs that are nearest neighbors (1-NN), i.e., one turn apart. The key idea behind the no-overlap constraint from Ref. [8] is to exploit the fact that whenever there is an overlap between two amino acids, there must also be an inter-peptide interaction from its bonded nearest neighbors. For any non-bonded pairs i, j a term is introduced that is equal to the MJ interaction energy ϵ_{ij} if a pair i, j is one edge apart and the pair's bonded particles do not violate an overlap constraint, and is strictly positive otherwise. This term will be multiplied with a binary interaction variable

$\omega_{ij} \in \{0, 1\}$, which is acting as a switch in the minimization, i.e, set to 0 if the term is positive and 1 if negative. In the energy minimization search, this will prefer the conformations without overlaps, as only interaction energy is counted in those cases.

To properly account for main- and side-chain interactions, the combined Hamiltonian is decomposed into three terms

$$H_{\text{comb}} = H_{\text{mm}} + H_{\text{ms}} + H_{\text{ss}} , \tag{S6}$$

where H_{mm} contains the terms associated with a main chain-main chain (main-main), H_{ms} main-side and H_{ss} side-side interaction pairs, respectively.

We first consider the main-main interaction term and note that because of the tetrahedral geometry, two main-chain particles A_i and A_j can only be 1-NN if they are an odd number of turns apart and $|j - i| \geq 5$. The Hamiltonian in Ref. [8] is given by

$$H_{\text{mm}} = \sum_{j \geq i+5\,, (j-i) \text{ odd}} \omega_{ij}(\epsilon_{ij} + \lambda_1(d(A_i, A_j) - 1) + \lambda_2 K_{ij}) , \tag{S7}$$

where $\lambda_1 > 0$ and $\lambda_2 > 0$ are penalty parameters, K_{ij} are the constraint penalty terms for the overlap checks on A_i with the 1-NN of A_j and A_j with the 1-NN of A_i

$$K_{ij} = \sum_{X \in N(A_j)} (2 - d(A_i, X)) + \sum_{X \in N(A_i)} (2 - d(X, A_j)) . \tag{S8}$$

Here, $N(A_i)$ denotes all the main and side chain particles bonded to A_i (e.g., $N(A_i) = \{A_{i-1}, A_{i+1}, A_i^s\}$ if $i \neq 1, N$ and A_i has side chain).

Note that whenever A_i and A_j are 1-NN, $\lambda_1(d(A_i, A_j) - 1)$ evaluates to 0. Moreover, each neighbor X of A_j either overlaps with A_i and $d(A_i, X) = 0$ or is two turns apart from A_j and $d(A_i, X) = 2$. Because two turns in the same direction are backtracking, the distance of two lattice sites that are two turns apart can only be 2 and not 4. In the case of no overlaps of the neighboring particles, we find $K_{ij} = 0$, yielding $\omega_{ij}\epsilon_{ij}$, which leads to the desired interaction energy ϵ_{ij} when minimizing. If there is an overlap, we have $K_{ij} > 2$, and by choosing $\lambda_2 > -\epsilon_{ij}/2$ for all i, j, we can ensure that the contribution is strictly positive and that ω_{ij} is set to 0 when minimizing.

If A_i and A_j are not 1-NN, then $\lambda_1(d(A_i, A_j) - 1) > 1$ because $d(A_i, A_j) \geq 2$. By choosing λ_1 large enough to compensate for the negative terms from K_{ij}, the term inside the summation is strictly positive. The bound for λ_1 has been given in Ref. [8] and requires that $\lambda_1 > 6(j - i + 1)\lambda_2 + \epsilon_{ij}$ for all i, j.

The same approach is applied to interaction pairs of main- and side-chain particles. We note that for A_i and A_j^s to be 1-NN, $|i - j|$ has to be even and at least 4 turns apart, leading to the expression

$$H_{\text{ms}} = \sum_{j \geq i+4\,, (j-i) \text{ even}} (h^{\text{mcsc}}_{ij^s} + h^{\text{mcsc}}_{ji^s}) , \tag{S9}$$

where

$$h^{\text{mcsc}}_{ij^s} = \omega_{ij^s}(\epsilon_{ij} + \lambda_1(d(A_i, A^s_{j^s}) - 1) + \lambda_2 K_{ij^s}) , \tag{S10}$$

and

$$K_{ij^s} = (2 - d(A_i, A_j)) + \sum_{X \in N(A_i)} (2 - d(X, A^s_{j^s})) . \tag{S11}$$

Compared to main-chain particles, where we have at most three 1-NN bonded neighbors $(N(A_i) \leq 3)$, side-chain particles have only one 1-NN bonded neighbor, i.e., the corresponding main chain particle, thereby reducing the number of terms in K_{ij^s} compared to K_{ij}. And while the bound on λ_2 does not change, the bound on λ_1 updates to $\lambda_1 > 4(j - i + 2)\lambda_2 + \epsilon_{ij}$ for all i, j because we consider one side-chain particle.

Finally, the side-side chain interaction term is given by

$$H_{\text{ss}} = \sum_{j \geq i+3\,, (j-i) \text{ odd}} \omega_{i^s j^s}(\epsilon_{ij} + \lambda_1(d(A^s_{i^s}, A^s_{j^s}) - 1) + \lambda_2 K_{i^s j^s}) , \tag{S12}$$

where

$$K_{i^s j^s} = (2 - d(A^s_{i^s}, A_j)) + (2 - d(A_i, A^s_{j^s})) . \tag{S13}$$

Here, we find that $\lambda_1 > 2(j - i + 3)\lambda_2 + \epsilon_{ij}$. The number of required interaction variables is therefore given by

$$N_\omega = \frac{(N-5)(N-4)}{4} + \frac{(N-4)(N-3)}{2} + \frac{(N-3)(N-2)}{4} \tag{S14}$$

and scales $\mathcal{O}(N^2)$, which is why the approach was referred to as "resource efficient" in Ref. [8].

C. Discussion of H_{protein} and derivation of bounds for penalty parameters

Let us discuss the different terms in H_{protein} and ensure that the Hamiltonian satisfies the necessary requirements. Starting with Eq. (3), we consider the relationship between a main chain particle A_i and a protein interaction site t_l^I. We consider the situation where A_i is located on the interaction site t_l^I. In this case, $d(A_i, t_l^I) = 0$ and we find that the first term reduces to ϵ_{il}. If all bonded 1-NN of A_i are not on a blocking or invalid site, we have that any pair (X, Y) in the sums in the second term are two turns apart and $d(X, Y) = 2$. Hence, the term associated with μ_2 vanishes and we end up, as required, with ϵ_{il}. For any bonded 1-NN of A_i on a blocking or invalid site, the distance $d(X, Y)$ evaluates to zero and we add a contribution $2\mu_2$. Hence, by choosing $\mu_2 > -\epsilon_{il}/2$ for all i, l, we ensure that the term associated with η_{il} in Eq. (3) is strictly positive.

Now let us consider the situation where A_i is not located on the interaction site t_l^I and show that the term associated with η_{il} is strictly positive. We have that the first term is strictly positive whenever $\mu_1 > 1$ because $d(A_i, t_l^I) \geq 1$ and $\epsilon_{il} < 0$. Next, we note that on the other side the term associated with μ_2 can be negative. Hence, we have to ensure that μ_1 is large enough to compensate any negative contribution of the second term. It is sufficient to choose $\mu_1 \geq 1 + 9\mu_2|N(A_i)||N(t_l^I)|/|\epsilon_{il}|$, where $|\cdot|$ applied to sets denotes the cardinality. Knowing that $|N_i| \leq 3$ and $|N(t_l^I)| \leq 3$, we can set $\mu_1 = \mu_2 * 81/|\epsilon_{il}| + 1$ for all i, l. With proper setting of μ_1 and μ_2, we are confident in the handling of the main-chain particles.

Rather than repeat the above analysis for the side-chain particles A_i^s, we just note that the same logic applies, but with a small change. That is that the check on the 1-NN neighborhood reduces down to checking the appropriate main chain particle A_i against the blocked and invalid sites, meaning only one summation across $M_{A_i}(t_l^I)$ is needed. With this, we obtain the expression in Eq. (4). Finally, the term in Eq. (5) ensures that if for any given interaction site t_l^I more than one associated interaction variable is switched on and set to 1, a penalty term proportional to μ_3 is added. Hence, by setting $\mu_3 > 0$ large enough, multiple interaction energies from a single interaction site are penalized and suppressed in the minimization. In conclusion, we have found that the term H_{protein} with the right choice for μ_1, μ_2 and μ_3 as given above satisfies the required properties.

It is left to show that the bound

$$\mu_1 \geq 1 + 9\mu_2|N(A_i)||N(t_l^I)|/|\tilde{\epsilon}_{il} \tag{S15}$$

ensures that the terms of H_{protein} in Eq. (3) proportional to the interaction variables η_{ij} are strictly positive whenever A_i is not on lattice site t_l^I. Hence, we require that for any fixed i, l

$$\tilde{\epsilon}_{il}(1 - \mu_1 d(A_i, t_l^I)) + \mu_2 \sum_{X \in N(A_i), k} [2 - d(X, t_k^B)] \geq 0\,. \tag{S16}$$

Solving the equation for μ_1, given that $d_0 := d(A_i, t_l^I)) > 0$ leads to

$$\mu_1 \geq \frac{1}{d_0} + \frac{\mu_2}{|\tilde{\epsilon}_{ij}| d_0} \sum_{X \in N(A_i), k} [d(X, t_k^B) - 2]\,. \tag{S17}$$

Because $d_0 \geq 1$, the first term in Eq. (S17) can be upper bounded by 1. To upper bound the second term, we note that any X is exactly one turn away from A_i so that its four-dimensional lattice turn representation can be written as $x(A_i) + y$ with $x(A_i)$ given in Eq. (S1), and y a single turn vector with exactly one component equal 1 and else 0. Similarly, any t_k^B can be written as $x(t_l^I) + y'$ with y' a single turn vector. Hence, we can bound

$$\begin{aligned}
d(X, t_k^B) &= \sum_i (x(A)_i - x(t_l^I)_i + y_i - y_i')^2 && \text{(S18)}\\
&\leq \sum_i (x(A)_i - x(t_l^I)_i)^2 + 2\sum_i (x(A)_i - x(t_l^I)_i)(y_i - y_i') + \sum_i (y_i - y_i')^2 && \text{(S19)}\\
&\leq d_0 + 2\sqrt{d_0}\sqrt{d(y, y')} + d(y, y') && \text{(S20)}\\
&\leq d_0 + 4\sqrt{d_0} + 4 && \text{(S21)}
\end{aligned}$$

using the Cauchy-Schwarz inequality in the third inequality and $d(y, y') \leq 4$ in the last step. Using this inequality, we can upper bound the right hand side of Eq. (S17) by $1 + 9|N(A_i)||N(t_l^I)|\mu_2/|\tilde{\epsilon}_{ij}|$ using that $d_0 \geq 1$. Therefore, choosing μ_1 larger than the derived upper bound suffices to ensure positivity in Eq. (S16) if $d(A_i, t_l^I) > 0$.

D. QUBO and CP solvers

D.1. QUBO Reduction

The problem Hamiltonian constructed in Manuscript Section 2 defines an unconstrained binary optimization problem of typically high polynomial order, which we will refer to as a PUBO (polynomial unconstrained binary

optimization). The higher-order terms are an issue, as current quantum annealing devices (e.g., D-Wave) do not allow for multi-body interactions. Rather, all terms must be quadratic [5]. Because our goal is to construct a solution to the problem that is quantum-amenable—meaning in this case that it could be run on a quantum annealing machine—we must address this restriction. To this end, we employ a technique known as locality reduction by substitution, wherein an additional variable is introduced and used to replace a two-body interaction term. However, this comes at a cost: we must also introduce a constraint that this new variable (bit) is equivalent to the product of the pair of variables (bits) it replaced. This necessarily adds more variables and terms to the problem Hamiltonian, although those terms are quadratic at maximum. For each pair of original bits to be replaced q_i and q_j, we introduce an ancilla bit q_{red}, adding a penalty term E_{red} as

$$E_{\text{red}}(q_i, q_j, q_{\text{red}}, P_{\text{red}}) = P_{\text{red}}(3q_{\text{red}} + q_i q_j - 2q_i q_{\text{red}} - 2q_j q_{\text{red}}), \tag{S22}$$

where P_{red} is a penalty weight. We can see that $E_{\text{red}} = 0$ if $q_{\text{red}} = q_i q_j$ and $E_{\text{red}} = P_{\text{red}}$ if $q_{\text{red}} \neq q_i q_j$. The choice of P_{red} is important, as it must be sufficiently large to prohibit a ground state where $q_{\text{red}} \neq q_i q_j$. This can be tuned carefully according to the problem at hand, or it can be set to cover a worst-case where somehow all ancilla bits q_{red} are misaligned. In this case, the energy of the Hamiltonian is shifted by as much as the aggregate terms in the expression. We aligned to this heuristic, and chose our bound as

$$P_{\text{red}} = \sum_{i,j} |Q_{ij}|. \tag{S23}$$

The optimal choice of substitution bits is an NP-hard problem, and so we leverage existing heuristic-based methods found in the dimod python library [3]. After applying locality reduction, the resulting Hamiltonian is of quadratic order, and is ready to be passed to a solver, such as a simulated or quantum annealing.

D.2. Hybrid simulated annealing solver

Simulated Annealing (SA) typically refers to Generalized Simulated Annealing, which combines Classical Simulated Annealing and Fast Simulated Annealing into one optimized framework [11]. SA is a probabilistic optimization technique useful in solving binary optimization problems, which is what we face here. In brief, SA operates iteratively over a number of temperature steps $t \in [T_{max}, T_{min}]$. For any given state (e.g., solution string) s, a neighbor state s' is identified, for instance by flipping a bit in s. The energies E of these two states are calculated, and the move to s' is accepted if the move is an improvement $[E(s') < E(s)]$, or it is accepted with a temperature-dependent probability $P[E(s), E(s'), t]$ if the move is not an improvement $[E(s') \geq E(s)]$. This probability function $P(\cdot)$ is expected to decrease towards zero as $t \to 0$, at which point the move is only accepted if it is an improvement, turning the algorithm into a greedy descent. By allowing for moves that do not improve performance, SA provides a mechanism to (potentially) escape local minima, which is a powerful ability in complex optimization energy landscapes. In this work we leverage the SA solver found in the D-Wave hybrid python library

After SA finds an updated state to the full problem, the state is passed to a tabu search solver for additional refinement. This tabu solver runs what is effectively a greedy neighborhood descent algorithm, taking steps from $s \to s'$ when $E(s') < E(s)$ if those proposed steps s' are not in the tabu list [12]. The generation of proposed steps s' may vary, but typically are individual or sets of bit flips on variables in s. The tabu list is a fixed sized list of previous visited states s', used to prevent immediate state cycling. States in the tabu list are removed after some number of steps (i.e., the tenure length) to allow the algorithm to revisit them. We use the tabu search implementation found in the D-Wave hybrid python library, which is based on Ref. [13]. After the tabu search has converged on a state, the loop is restarted and the SA solver starts again.

D.3. QUBO decomposition

While quantum devices, like the D-Wave Advantage series [5], may house hundreds or thousands of physical qubits, these qubits are not densely connected on the device, and so often multiple physical qubits must be networked together in order to establish long-range connections [14]. This means that individual problem variables (e.g., q_1) must be mapped to sets of physical qubits. As the problem density (connectivity) increases, more physical qubits must be consumed to enable connectivity, and so fewer problem variables can be mapped onto the device at one time. If we want to solve large problems, say with hundreds of variables, we will need some mechanism external to the quantum device itself to overcome this limitation.

One way to achieve this is via problem decomposition: Break the full QUBO problem into sub-problems of a given fixed size k, solve for the variables in this sub-problem while keeping the other variables fixed, evaluate the newly-generated state and keep it if it is an improvement, then step to the next sub-problem and repeat until convergence [15]. The choice of sub-problems is key to the success of problem decomposition, and of course there exist numerous generalized options. For instance, one could choose a starting state $\mathbf{q}$ and estimate the energy impact of each variable q_i by independently flipping each of their values and measuring the change in system energy. This creates a mapping of each variable q_i to its measured energy impact on the system,

conditioned on the state $\mathbf{q}$. One could then sort the list by descending energy impact and select out k variables at a time to solve for. This is the default behavior of D-Wave hybrid's `EnergyImpactDecomposer` object, and is the decomposition strategy we use in this work. Typically these decomposition strategies assume each variable q_i is included exclusively in a single sub-problem, and once all variables in $\mathbf{q}$ have been explored (one full sweep), the process restarts based on the latest best state $\mathbf{q'}$; typically $\mathbf{q} \neq \mathbf{q'}$, else no progress had been made on that sweep.

E. Resource scaling analysis

As part of our initial exploration into solving the peptide docking problem, we have implemented multiple problem formulations in parallel. Spatial encoding (see Supplementary F), the turn ancilla encoding (see Supplementary G), and the variable efficient turn encoding (see Manuscript Section 2). Our goal has been to primarily understand the details of each approach, and also to explore which approach would be able to handle the full peptide and protein active site inclusion. We have implemented feature-complete solutions for each, including all problem constraints and the external protein. We assess the scalability of these approaches by taking a particular problem instance (in this case, PDB file 2CK0), down-sampling the peptide and protein residues, constructing Hamiltonians for each approach on the down-sampled problems, and measuring the number of variables and terms required for the polynomial unconstrained binary optimization (PUBO) and QUBO Hamiltonians. We stepped across peptide sizes $N \in [4, 10]$ and $M \in [0, 10]$, with $N = 4$ being the minimum length required for a complete cycle (assuming side chains exist) and $N = 10$ being the full length of the peptide, and M being the number of protein residues included (with $M = 0$ meaning the protein was excluded). Note that we did not need to include the full set of protein residues to be able to draw conclusions from this analysis.

Figure S1 shows the results of this analysis. We see that the spatial QUBO (SQ) approach is unable to scale beyond $N = 5$. Interestingly, while the number of variables required in the SQ PUBO is the smallest for all approaches, the number of variables in the QUBO is the highest. This indicates that the SQ Hamiltonian contains terms of very high order, which generate significant overhead in the reduction to a QUBO formulation. It is expected that reduction from PUBO to QUBO introduces some amount of overhead, in particular in terms of the number of variables in the expression (see Supplementary D), but it is clear that the SQ approach suffers significantly more from this effect than the resource efficient (RE) or turn ancilla (TA) approaches. We note that the RE approach—true to its name—is in fact more efficient that either the SQ or TA approach, and this holds across the range of values of N and M tested. There is a discontinuity in the number of terms (although less apparent at high M) at $N = 6$, which is the first length where the main-main cyclization constraint could be enforced, thus adding additional terms to the Hamiltonian.

F. Spatial formulation

The spatial encoding on the tetrahedral lattice $\mathcal{T}$ is derived from the original two-dimensional (2D) square grid formulation presented in Ref. [1]. The three-dimensional (3D) tetrahedral lattice is considered a special case of the 3D cubic lattice, where atoms are placed on only opposite corners of each face of a cube, and not all vertices are occupied. Here, we adopt and extend the spatial encoding as described in Ref. [1], modifying the Hamiltonian to penalize all the 3D cubic vertices that cannot be occupied so that the resulting lattice is tetrahedral.

We consider a two-particle (main and side chain) coarse grained (CG) model for each amino acid except for smaller amino acids e.g., glycine, which is represented using one-particle (main chain) coarse grained model. The position of each of $N + N^s$ particles (sum of main and side chain) in a D-dimensional cubic lattice can be encoded by $D(N + N^s)\log_2(N + N^s)$ binary variables. We used the same distance function as described in Eq. (22) in Ref. [1] to represent the rectilinear distance (L1) squared between grid points. Note that this distance squared is equal to 1 in square and cubic lattices but is equal to 3 for the tetrahedral lattice.

Here we describe the logic more symbolically than in Ref. [1], imagining there is a function $f(*)$ that takes in a peptide particle i (or an equivalent protein-influenced vertex $t_l^I \in \mathcal{T}_{\mathcal{I}}$), and retrieves the bits describing the location of that particle in grid coordinates, in an ordered fashion such that the bit strings of multiple particles $f(i)$ and $f(j)$ can be compared directly.

F.1. Hamiltonian construction

We start our Hamiltonian formulation based on the simplified approach for a 2D grid lattice of one-particle peptide sequence as described in Ref. [1], without cyclization and peptide-protein interaction terms. The Hamiltonian is given by

$$H = H_{\text{onsite}} + H_{\text{psc}} + H_{\text{pairwise}}, \tag{S24}$$

where H_{onsite} is an onsite repulsion term for amino acids occupying the same grid point, H_{psc} is a primary sequence constraint term, and H_{pairwise} is a pairwise interaction term that represents favorable hydrophobic

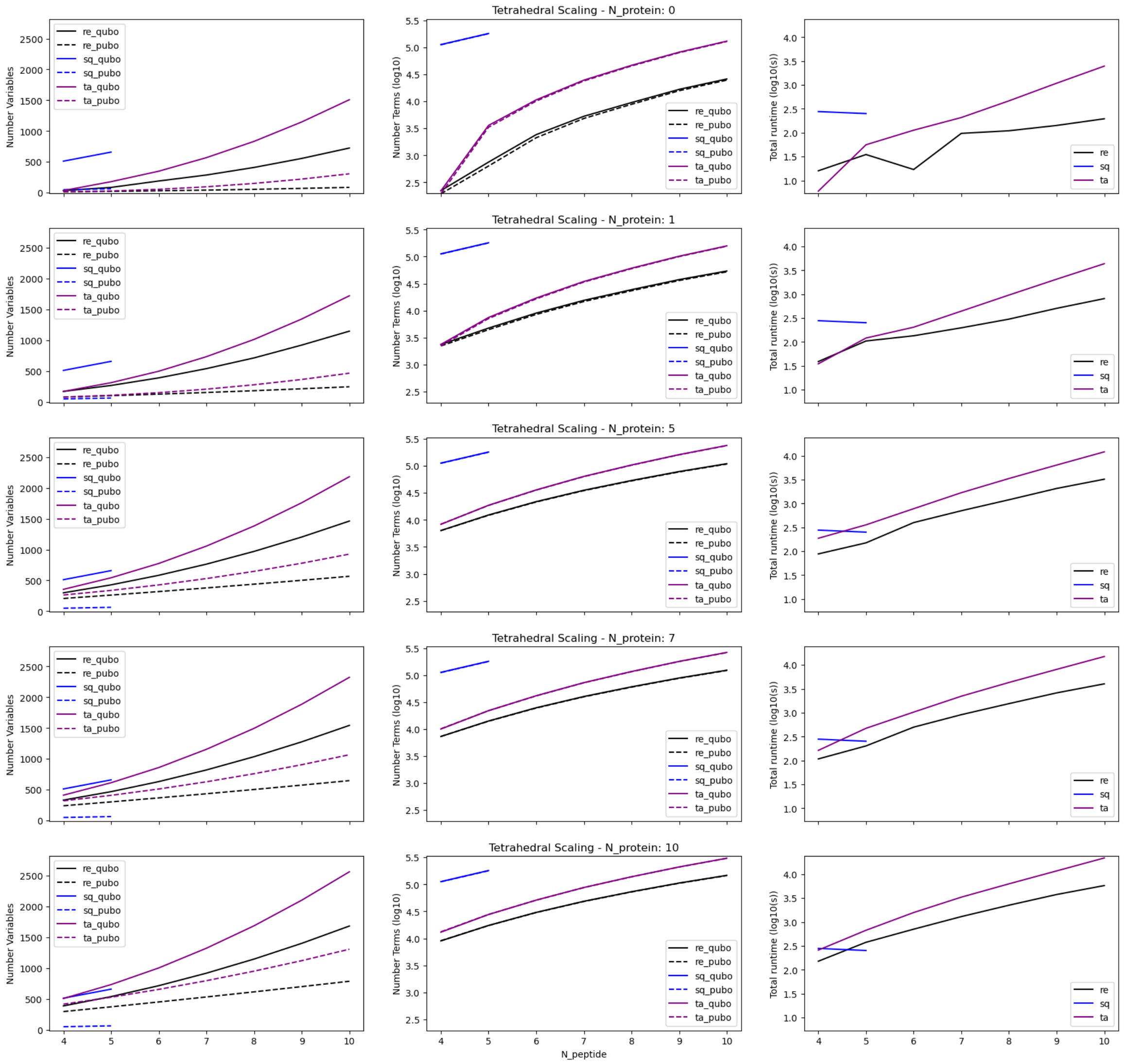

Figure S1: Problem scaling for each approach: spatial QUBO (SQ), turn ancilla turn encoding (TA), and resource efficient turn encoding (RE) as a function of the peptide length for polynomial (PUBO) and quadratic (QUBO) Hamiltonian forms. Number of protein residues included starts at $M = 0$ (top row) and increases until $M = 10$ (bottom row). (left) Number of variables required for the Hamiltonian for each approach. (middle) Number of terms ($\log_{10}$) required for the Hamiltonian for each approach. (right) End-to-end runtime for each approach.

interactions between adjacent hydrophobic amino acids. Note that original formulation was derived for HP (Hydrophobic-Polar) interactions between amino acids. We modify the Hamiltonian and included additional terms, as follows:

$$H = H_{\text{onsite}} + H_{\text{psc}} + H_{\text{pairwise}} + H_{\text{cycle}} + H_{\text{extpairwise}} + H_{\text{extonsite}} + H_{\text{invalid}}, \tag{S25}$$

where H_{cycle} is a cyclization constraint term, $H_{\text{extpairwise}}$ is a pairwise interaction term between external protein and peptide, and $H_{\text{extonsite}}$ is an onsite (steric blocking) constraint term between external protein and peptide.

F.1.1. Overlap constraint penalty term

We use Eq. (20) in Ref. [1] as the first term in Eq. (S25) to prevent two or more particles (main or side chain) from occupying the same grid point. Symbolically, we can describe the logic as:

$$H_{\text{onsite}} = \lambda_0 \sum_{ij} \prod_{k} \text{XNOR}]f(i)_k, f(j)_k], \tag{S26}$$

where XNOR is the exclusive NOR operator, returning 1 if both bits are equal, and 0 otherwise. In other words, this term steps through all bits in $f(i)$ and $f(j)$ and compares them, and if any bits do not match, the inner product returns 0, otherwise it returns 1, thereby signaling an overlap. This is repeated for all pairs of peptide particles i, j, the overlaps are summed, and a penalty of λ_0 is applied.

F.1.2. Primary sequence constraint penalty term

Extending to a two-particle model requires updating H_{psc} from the original implementation to include the distance requirement between between main and side chains of the same amino acid, i.e.,

$$H_{\text{psc}} = \lambda_{\text{psc}} \left[d_{ij}^2 - d'((N + N^s) - 1) \right], \tag{S27}$$

where λ_{psc} is a strictly positive scalar to regulate the penalty strength, $(N + N^s)$ is the number of main- and side-chain particles, and d' is the distance cutoff of a specific lattice type ($d' = 3$ for tetrahedral and $d' = 1$ for the square or cubic lattice), and d_{ij}^2 represents the squared rectilinear distance between particles i and j.

F.1.3. Pairwise interaction term

We extend H_{pairwise} to include a MJ potential [16] and modify the formulation based on the distance between any two particles on the grid. The non-bonded amino acid particles that are one bond distance square apart can have pairwise interactions. In 2D or 3D, one bond distance squared is given by one rectilinear unit distance squared but for the tetrahedral geometry, one bond distance squared is given by 3 rectilinear units distance squared. The term is given by:

$$H_{\text{pairwise}} = \sum_{ij} \Omega_{ij} \left[G_{ij} * (d' - d_{ij}^2) \right], \tag{S28}$$

where Ω_{ij} is an ancillary bit to turn on or off the potential pairwise interaction between particles i and j. G_{ij} is the interaction (MJ) energy, d' is the distance cutoff for a specific lattice type ($d' = 4$ for tetrahedral and $d' = 2$ for square or cubic lattices), and d_{ij}^2 represents the rectilinear distance squared between particles i and j.

F.1.4. Cyclization constraint penalty term

Next, we add a penalty term H_{cycle} that enforces the cyclization bond C between given peptide residue pairs i and j. We enforce this constraint by forcing the particles of the residue i and j to be nearest neighbors on the lattice. This can be enforced by a penalty term of the form

$$H_{\text{cycle}} = \lambda_{\text{cycle}} \sum_{ij} (d_{ij}^2 - d'), \tag{S29}$$

where λ_{cycle} is a strictly positive scalar to regulate the penalty strength and d' is the distance cutoff of a specific lattice type ($d' = 3$ for tetrahedral and $d' = 1$ for square or cubic lattices), and d_{ij}^2 represents the rectilinear distance square between particles i and j. H_{cycle} is equal to zero if the particles are one lattice step apart and adds a positive contribution otherwise. By choosing λ_{cycle} appropriately, we can ensure that the minimization leads to a state satisfying the cyclization constraint.

F.1.5. Protein interaction terms

Next, we follow a similar protocol for the protein-peptide interaction as described in Manuscript Section 2. However, we first must project the external protein influence onto the lattice vertices and calculate the proper set of influenced coordinates $\mathcal{T}_{\mathcal{I}}$. We first introduce the pairwise interaction term between the peptide and external protein:

$$H_{\text{extpairwise}} = \sum_{i,l} \left[\Omega_{il} G_{il} (d' - d_{it_l^{\mathcal{I}}}^2) \right], \tag{S30}$$

where Ω_{il} is an ancillary bit to turn on or off the potential pairwise interaction between peptide particle i and protein interaction-influenced lattice vertex $t_l^{\mathcal{I}} \in \mathcal{T}_{\mathcal{I}}$. G_{il} is the MJ interaction potential, d' is the distance cutoff of specific a lattice type ($d' = 4$ for tetrahedral and $d' = 2$ for square or cubic lattices), and $d_{it_l^{\mathcal{I}}}^2$ represents the rectilinear distance squared between i and $t_l^{\mathcal{I}}$.

Next we introduce the onsite term between the peptide and external protein, penalizing overlaps. We again do this on the symbolic level, as we did with Eq. (S26), although here we check peptide particles i against protein blocked sites $\mathcal{T}_{\mathcal{B}}$:

$$H_{\text{extonsite}} = \lambda_0 \sum_{il} \prod_{k} \text{XNOR} \left[f(i)_k, f(t_l^I)_k \right], \tag{S31}$$

again using XNOR checks across the bit strings representing the lattice locations of peptide i and protein-blocked site $t_l^B \in \mathcal{T}_{\mathcal{B}}$. Note that we use the same overlap penalty term λ_0.

F.1.6. Invalid grid points penalty term

Finally, we need to add an additional Hamiltonian term to penalize the invalid positions on a cubic grid that are not part of the tetrahedral lattice. Invalid lattice coordinates are penalized by running overlap checks between positions of all particles and invalid lattice coordinates. Symbolically, we can establish the set of valid lattice coordinates, which is equivalent to the tetrahedral lattice set introduced in Manuscript Section 2, $\mathcal{T}$, the full set of cubic lattice coordinates as $\mathcal{T}_{cubic}$, and the set of invalid lattice coordinates as

$$\mathcal{T}_{\text{invalid}} = \mathcal{T}_{cubic} \setminus \mathcal{T}. \tag{S32}$$

With these set definitions, we can check for invalid grid placements by counting the overlaps between each peptide particle a_i and $\mathcal{T}_{\text{invalid}}$, as follows

$$H_{\text{invalid}} = \lambda_{\text{invalid}} \sum |a_i \cap \mathcal{T}_{\text{invalid}}| \quad \forall a_i \in A \cup A^s. \tag{S33}$$

Note that the choice of λ_{invalid} is described below. In reality, we check the bit strings representing the coordinates in a_i and $\mathcal{T}_{\text{invalid}}$, but this is a matter of mapping from the coordinates to the bit strings, so the logic remains consistent.

F.2. Choice of Penalty terms

We have seven terms in the final Hamiltonian in Eq. (S25) including pairwise functions. H_{pairwise} and $H_{\text{extpairwise}}$ are MJ interaction terms, which the model seeks to minimize, as the MJ values are negative. We assume the same penalty λ_0 for H_{onsite} and $H_{\text{extonsite}}$ as they represent similar overlap constraints, and the same penalty λ_1 for H_{psc} and H_{cycle} as they represent similar sequence constraints. We use λ_{invalid} to represent the penalty term for invalid grid points in H_{invalid}. The choice of penalty terms requires some considerations to achieve an optimal solution using classical simulated annealing solvers.

First, let us consider onsite (penalty) and pairwise (favored) interactions. For the square grid, the onsite penalty must be more than three times the maximum of the MJ potential, i.e., $\lambda_0 > 3 \times 7.73 \approx 23$. For the cubic lattice, the onsite penalty must be more than five times the maximum of the MJ potential, i.e., $\lambda_0 > 37$. Finally, for the tetrahedral lattice we need to set the onsite and invalid lattice site penalty to be at least five times the maximum of the MJ potential, i.e., $\lambda_0 = \lambda_{\text{invalid}} > 37$. The primary sequence penalty term is given by $\lambda_{\text{psc}} = -(n-1) + d_{ij}^2$ for the square and cubic lattices. For the tetrahedral lattice we use $\lambda_{\text{psc}} = -3(n-1) + d_{ij}^2$.

If all particles were placed on top of each other (i.e., the extreme case) the bit strings would either be $[0, 0, 0, \ldots]$ or $[1, 1, 1, \ldots]$. The number of onsite violations is therefore $n!/(n-2)! = n(n-1)/2$. Because in this case $d_{ij}^2 = 0$ for all i and j, the number of primary sequence violations is $(n-1)$. As such, $\lambda_0 n(n-1)/2$ must be larger than $\lambda_1(n-1)$. In this work we treat the primary sequence and cycle penalties in the same way, i.e., if all particles would be restricted to one lattice vertex there will be favorable energy from H_{cycle}. For the square and cubic lattices this energy is $(d_{ij}^2 - 1)$ and for the tetrahedral lattice $(d_{ij}^2 - 3)$. Therefore, $\lambda_0 n(n-1)/2$ must be larger than $\lambda_1(n-1+1)$. This is the extreme case for the 3D lattice. For the tetrahedral lattice, $\lambda_0 n(n-1)/2$ must be more larger than $3\lambda_1(n-1+1)$. Setting $n = 2$, we obtain $\lambda_0 > 6\lambda_1$. It is probably safe to set $\lambda_0 = 10\lambda_1$. However, because $\lambda_0 > 37$, $\lambda_1 \geq 4$. Finally, if we consider the onsite penalty and invalid site penalty for the tetrahedral lattice, one option is to keep $\lambda_0 = \lambda_{\text{invalid}}$. However, to avoid violations we set $\lambda_{\text{invalid}} = 2\lambda_0$. Summarizing, we use the following values for the different penalty terms: $\lambda_1 = 4$, $\lambda_0 = 10$ $\lambda_1 = 40$, and $\lambda_{\text{invalid}} = 2$ $\lambda_0 = 80$.

G. Turn ancilla formulation

Here we describe an alternative turn encoding approach—the turn ancilla encoding—which is an extension of the work of Babbush *et al.* in Ref. [3]. We seek to formulate the problem described as a PUBO, which can then be reduced to a QUBO and solved using an optimizer such as simulated annealing.

The turn encoding defines the peptide conformation by a series of turns, either along the main-chain backbone, or off of it to place side-chain particles, starting from the first main-chain residue (assumed to be placed at the lattice origin). The tetrahedral lattice has 4 possible turns from any given vertex, which are counter-directional on alternating sublattices: $t \in (0, 1, 2, 3)$ on $\mathcal{T}_{\mathcal{A}}$ and $t \in (\bar{0}, \bar{1}, \bar{2}, \bar{3})$ on $\mathcal{T}_{\mathcal{B}}$. We leverage a bit mapping that maps $(0, 1, 2, 3) \mapsto (00, 01, 10, 11)$ on $\mathcal{T}_{\mathcal{A}}$, and $(\bar{0}, \bar{1}, \bar{2}, \bar{3}) \mapsto (00, 01, 10, 11)$ on $\mathcal{T}_{\mathcal{B}}$. For a peptide sequence A, we require $|A| - 1$ turns for the main chain and $|A^s|$ turns for the side chain, which, requiring 2 bits per turn, means we need a total of $|q| = 2 * (|A| - 1 + |A^s|)$ bits to define the peptide conformation. Distance between particles on the lattice is defined by Eq. (S3). As a reminder, the distance between a particle and a lattice vertex can be calculated by the same equation, noting that in the case of a vertex, the turn vector x would represent the number of steps along each dimension k from the origin to that vector. With this in mind, we can now discuss the turn ancilla Hamiltonian construction.

G.1. Turn ancilla Hamiltonian

We start by reviewing the Hamiltonian terms introduced in Ref. [3], although we note that Babbush *et al.* built the peptides on a square grid whereas we are building these on a tetrahedral lattice. Thus, we update the definitions of these Hamiltonian terms accordingly

$$H = H_{\text{back}} + H_{\text{pair}} + H_{\text{overlap}}, \tag{S34}$$

where H_{back} is a special case of H_{overlap}, checking that sequential turns do not yield overlapping residues (i.e., backtracking), H_{pair} calculates the total MJ interaction potential between relevant residue pairs in the solution, and H_{overlap} calculates a penalty for residue pairs that overlap on the same lattice vertex. To define H_{back}, we use the helper function $T(t,t') = \sum_{k=0}^{3} f_k(t)f_k(t')$, which, again, is equal to 1 if turns t and t' are in the same direction and 0 otherwise. We can then build H_{back} using the helper function as follows:

$$H_{\text{back}} = \sum_{i=1}^{N-1} T(t_i, t_{i+1}) + \sum_{i=2}^{N-1} T(t_{i-1}, s_i) + \sum_{i=1}^{N-1} T(t_i, s_i)\,, \tag{S35}$$

which checks for backtracking on sequential main-chain turns, on the previous main-chain turn and current side-chain turn, and on the current main-chain turn and side-chain turn.

To understand H_{pair}, we note that g_{ij} in Eq. (S36) is used to represent the squared distance between residues i and j; in other words, it represents the value of Eq. (S3). As part of our formulation, we assume that only residue pairs at a distance of 1 (i.e., 1-NN) can interact with one another. Thus, we need an equation for each residue pair that returns a negative value (i.e., is beneficial) when $g_{ij} = 1$ and 0 otherwise. Noting that $g_{ij} \geq 0$, we can accomplish this via

$$H_{\text{pair}}(i,j) = \omega_{ij}\text{MJ}(i,j)(2 - g_{ij}), \tag{S36}$$

where ω_{ij} is the interaction ancilla for pair (i,j) and $\text{MJ}(i,j)$ is the Miyazawa-Jernigan potential between pair (i,j). If $g_{ij} = 0$, then we have an overlap between particles, which should be penalized via H_{overlap}, and if $g_{ij} > 2$ then $2 - g_{ij}$ is negative, and because $\text{MJ}(i,j)$ is always negative, the result is a positive value, which the solver should avoid. The interaction ancillas ω_{ij} are useful in preventing the solver from adding pairwise interaction terms between all main chain residues (i.e., residues that are connected by a turn), as those residues will always be a distance of 1 from each other. As such, ω_{ij} prevents the solver from interpreting these residue pairs as beneficial to the overall solution.

H_{overlap} penalizes any residue pairs that are at a distance of 0 (i.e., overlapping), but to do so in a manner that leverages a fixed number of variables, which can be specified prior to solving. To do this, rather than compare the distance between two residues g_{ij} against 0 (which is an unbounded comparison), we compare the distance against the upper bound of that distance $2^{mu_{ij}}$. This is why we require slack ancillas: We want to ensure the slack can compensate for distance up to $2^{mu_{ij}} - 1$, so that if $g_{ij} \geq 1$, then $H_{\text{overlap}} = 0$, else if $g_{ij} = 0$, then $H_{\text{overlap}} = 1$. We start top-down, specifying that for each potentially interacting residue pair i, j

$$\gamma_{ij} = (2^{\mu_{ij}} - g_{ij} - \alpha_{ij})^2, \tag{S37}$$

where $2^{\mu_{ij}}$ is the maximum possible squared distance between residues (i,j) (i.e., if they were in a straight line) and α_{ij} specifies the ancilla bits relating to residue pair (i,j). Note that

$$\alpha_{ij} = \sum_{k=0}^{\mu_{ij}-1} q_{c_{ij}+k} 2^{\mu_{ij}-(k+1)} \leq 2^{\mu_{ij}} - 1, \tag{S38}$$

where c_{ij} is a pointer to the correct starting bit for α_{ij}, which requires k bits, among all bits in the solution string. Finally, when applied to the potentially interacting residue pairs, we obtain

$$H_{\text{overlap}} = \sum \gamma_{ij} \tag{S39}$$

which returns the total number of overlaps, or 0 if there are none. This result is then scaled by a penalty weight λ_{penalty}.

G.1.1. Cyclization constraint term

We define the cyclization constraint as a constraint enforcing the distance between the residue pairs specified in C_{ij} to be equal to 1. In other words, $g_{ij} = 1 \forall i, j \in C$. Because we have a formula for the distance between any two residues, this constraint is easily implemented. We add a term to the system Hamiltonian H_{cycle}, which encodes this constraint:

$$H_{\text{cycle}} = \lambda_{\text{penalty}} \sum_{C} (g_{ij} - 1)^2, \tag{S40}$$

where $\lambda_{penalty}$ is a penalty weight. The choice of λ_{penalty} is important, because using a value that is too small may (and likely will) yield states that violate these constraints. However, values too large may prevent the solver from exploring infeasible regions on its way to finding potentially optimal regions. To find a minimum useful value for λ_{penalty}, we can calculate the energy of a state where all peptide residues occupy two lattice vertices, which would yield the maximal intra-peptide energy E_{peptide}. Alternatively, the peptide could form a straight, non-cyclized chain, such that it maximizes the peptide-protein interaction energy $E_{\text{peptide}-\text{protein}}$. These values vary depending on the problem configuration, but once calculated, we can consider setting $\lambda_{\text{penalty}} = \max\{|E_{\text{peptide}}|, |E_{\text{peptide}-\text{protein}}|\} + 1$. In practice, we find that setting the value much higher, for instance to $\lambda_{\text{penalty}} = 1000$ to be simpler and more useful to keep the model results from violating these constraints.

G.1.2. Protein interaction term

To derive the protein interaction term, we first project the protein particles' effect onto the lattice, yielding a set of blocked vertices $\mathcal{T}_{\mathcal{B}}$ and a set of interaction vertices $\mathcal{T}_{\mathcal{I}}$. In order for a peptide residue i to feel the effect of the protein, it must directly overlap with a projected protein vertex t_l ($d(i,l) = 0$). We expect that any interaction site t_l^I will have some number of 1-NN blocked sites t_l^B, and so if residue i does overlap with interaction site t_l^I, we must check for 1-NN blocking sites t_k^B. The Hamiltonian therefore is given by

$$H_{\text{protein}} = \sum_{i,l} \eta_{il} \big(\tilde{\epsilon}_{il} (1 - \lambda_1 d(A_i, t_l^I)) + \lambda_2 \sum_{X \in N(A_i),k} (2 - d(X, t_k^B)) \big) \tag{S41}$$

$$+ \sum_{i,l} \eta_{i^s l} \big(\tilde{\epsilon}_{il} (1 - \lambda_1 d(A_i^s, t_l^I)) + \lambda_2 \sum_k (2 - d(A_i, t_k^B)) \big) \, . \tag{S42}$$

For any peptide residue A_i and protein interaction site t_l^I, if $d(A_i, t_l^I) > 0$ then there is a positive energy contribution which should be turned off by $\eta_{i,l}$, else if $d(A_i, t_l^I) = 0$, then we perform 1-NN checks on $N(A_i)$ and $N(t_l^I)$ to confirm no peptide particles land on a blocking site. We chose to include the external protein interactions in this manner as opposed to continuing with the turn ancilla bits, because the variable scaling limitation of the turn ancillas before attempting to include the protein. As we can see in Supplementary E, the variable scaling of the resource efficient approach for peptide-peptide interactions is of the order $\mathcal{O}(N^2)$, whereas the variable scaling for turn ancilla is on the order of $\mathcal{O}(N^2 \log_2(N))$.

G.2. Complete Hamiltonian

Summing over all terms derived above, results in the following composite turn ancillary problem Hamiltonian:

$$H = H_{\text{pair}} + H_{\text{protein}} + \lambda_{\text{penalty}} (H_{\text{overlap}} + H_{\text{back}} + H_{\text{cycle}}). \tag{S43}$$

H. Additional experimental details

H.1. Experimental Setup

We have implemented the problem instance generation pipeline and the QUBO and CP solution approach in Python. For the QUBO approach, we use the dimod library from D-Wave Systems to perform the PUBO to QUBO locality reduction. We use the open source D-Wave Hybrid framework for the simulated annealing (SA) and decomposition-based experiments. For each problem instance (peptide), we run hyperparameter optimization (HPO) via Amazon SageMaker over the following parameters: λ_{cycle}, λ_{back}, μ_2, μ_3, p, *sub_qubo_size*, *num_reads*, *num_sweeps*. Because SA is a probabilistic algorithm, we run 10 shots for each of the problem instances on each fixed parameter solver run.

For the HPO algorithm, we use the SageMaker Tuning Job built-in Bayesian optimization, the details of which can be found in the SageMaker Documentation. Each step in the HPO process reports back only the best loss found across all 10 shots, and the optimization acts on that value. The loss of a given solution was defined as the Hamiltonian energy H evaluated at that solution, plus a scaled (1000x) count of the violations found in that solution. Each HPO step is allowed to run for up to 4 hours, and if that time is exceeded, the step is considered a failure and results are not reported. We limit the HPO search to 100 steps, truncating the run if the algorithm has not converged by then.

The CP model is implemented using the OR-Tools library [17] and uses all settings at their default values, except for the run-time limit, which is set to 300 seconds. The compute resources used for the experiments are Amazon Web Services Inc. (AWS) Elastic Cloud Compute (EC2) m5.4xlarge instances. These instances feature an Intel Xeon Platinum 8000 series processor, with 16 virtual CPUs and 64GB of memory.

The visualizations presented in this paper use a combination of the Mol* 3D viewer [18] available from the RCSB Protein Data Bank as well as 3dmol.js [19].

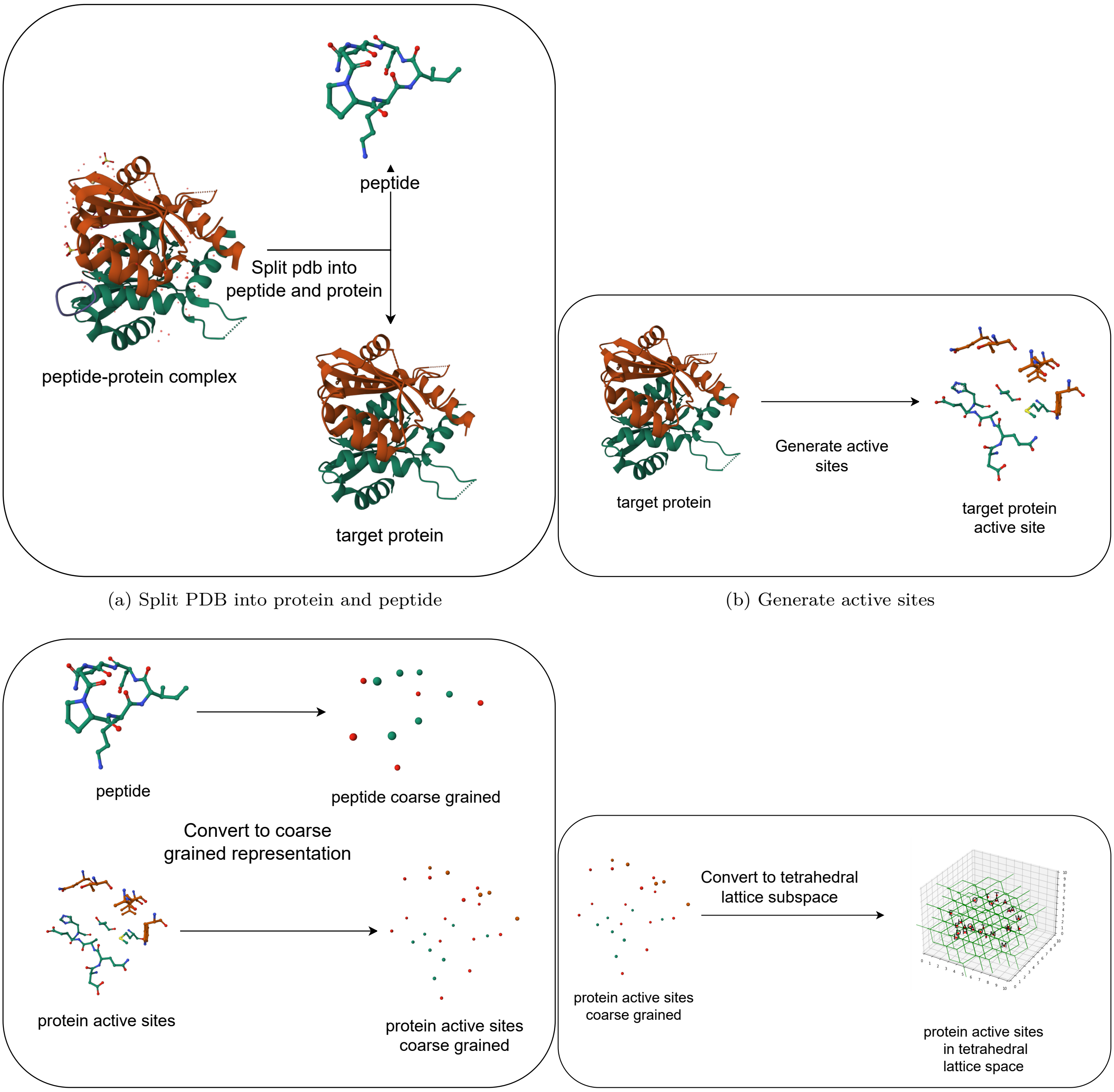

(a) Split PDB into protein and peptide

(b) Generate active sites

(c) Convert to coarse grained particles

(d) Construct tetrahedral lattice

Figure S2: Problem instance generation pipeline for peptide 3WNE from an atomic-level, continuous coordinate PDB representation to a 2-particle coarse grained tetrahedral lattice representation. Details are described in the main text.

H.2. Pipeline definition

Here we detail the pipeline defined in Manuscript Section 3 and outlined in Fig. S2. For all experiments we use a lattice distance of 3.8Å, and for the target protein interaction a blocking radius of 2.5Å to 3.5Å (steric hindrance), and MJ interaction potentials within a radius of 6.5Å. We note that our solution allows for different radii to be specified, so long as the interaction radius is at least 1.633 times larger than the blocking radius.

The first step of our approach is to separate the information in the PDB file into two components, namely the peptide, and the target protein (i.e., the protein that the peptide docks onto). Extracting these components is easily achieved using the information in the header of the PDB file. For protein 3WNE specifically, the header specifies that the PBD contains two types of molecules: A polyprotein (i.e., the target protein) and a synthetic peptide (i.e., the peptide we seek to dock on the target). Note that these two components are characterized by a series of coordinates specifying where each atom of the protein/peptide is located in space. The next step in the procedure is to identify the protein active site (Figure S2b), which represents a smaller, more focused area that the peptide is likely to use for docking. Determining an effective active site is important because smaller active sites result in fewer variables in the problem encoding, and thus a higher likelihood of being able to produce a solution. To isolate the active site we find the residues in the target protein that are less than some distance threshold (in this case, 5Å) from the peptide residues. Note that the specific distance threshold used impacts

Algorithm S1 Construct filtered tetrahedral lattice, $\mathcal{T}' = \{t_1, t_2, \dots\}$

Input: Target site centroid representation, P, lattice depth, δ, steric clash radius, α, shift step δ
Output: Filtered tetrahedral lattice, $\mathcal{T}'$, shifted target site coordinates, P

```
t_1 ← CENTROID(P)                  % determine the origin of lattice
T ← GROWLATTICE(t_1, δ)            % grow the lattice from the origin according to depth
T' ← T \ T_B                       % remove vertices affected by steric clash
while INVALIDLATTICE(T') do
    P ← SHIFT(P, δ)                % shift target site away from lattice origin
    T' ← T \ T_B                   % remove vertices affected by steric clash
end while
```

the size of the active site, and thus the quality of the solutions produced by our optimization approach.

With the target protein active site and peptide coordinates in hand, the next step is to calculate the coarse-grained 2-particle representation of each of the amino acids in these structures (Figure S2c). This is accomplished by classifying each of the atoms in each residue as main chain or side chain and then calculating the centroid (adjusted by molecular weight) of these groupings. The result is a two-particle representation of the amino acid, where each of the particles is characterized by a coordinate and an amino acid label, as described in Manuscript Section 2. Note that in the case of Glycine, only a single particle is generated because there is no side chain.

The last step in our problem instance generation procedure is to produce the tetrahedral lattice that discretizes the search space for the binary optimization approach (Figure S2d). Our approach for placing the tetrahedral lattice is described in Algorithm S1. We generate an initial candidate for the lattice origin by taking the centroid of the active site P and then grow the tetrahedral lattice recursively up to depth δ. In our implementation we enforce discrete-valued lattice vertices by rounding the origin coordinates to the nearest integer, and then using an edge length of $\sqrt{3}$ for the tetrahedral lattice. We assume that lattice edges correspond to a distance of 3.8Å, and so we scale the coordinates of the active site P accordingly (e.g., a protein particle with coordinate (1Å, 1Å, 1Å) in the original PDB file becomes $(\sqrt{3}/3.8, \sqrt{3}/3.8, \sqrt{3}/3.8)$ in our tetrahedral lattice scale). We estimate the required depth δ based on the distance from the selected origin to the furthest point in the active site. The lattice is then filtered according to the current active site coordinates P and a steric clash parameter α to remove vertices that would clash with the target site, $\mathcal{T}_{\mathcal{B}}$. A subroutine tests whether the resultant lattice is capable of producing a feasible conformation (e.g., the origin is checked for steric clash and evidence there is at least one route available to yield a cyclic conformation). If the lattice is invalid, the target site coordinates are shifted away from the origin of the lattice (by step size δ in the direction of the rest of the target protein) and the process repeats until a valid filtered lattice is determined.

H.3. Additional Solution Visualizations

We provide additional three-dimensional visualizations of CP and QUBO solutions for the remaining peptides in Table 1, to help give context to the results, in Figures S3, S4, S5, S6, S7.

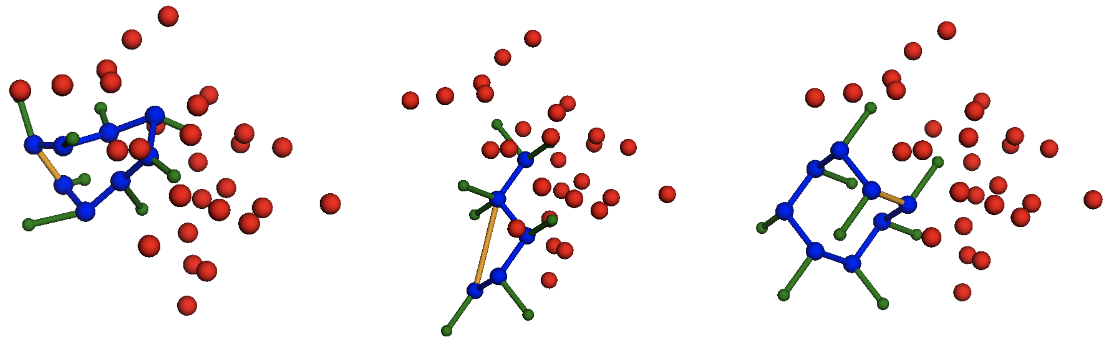

Figure S3: 3AVN result visualization. True PDB peptide (left) against the QUBO model result (middle) and the CP-MJ 1-NN result (right). The peptide is represented with blue and green dots (main and side-chain, respectively, conected by lines), and the red dots are the external protein residues.

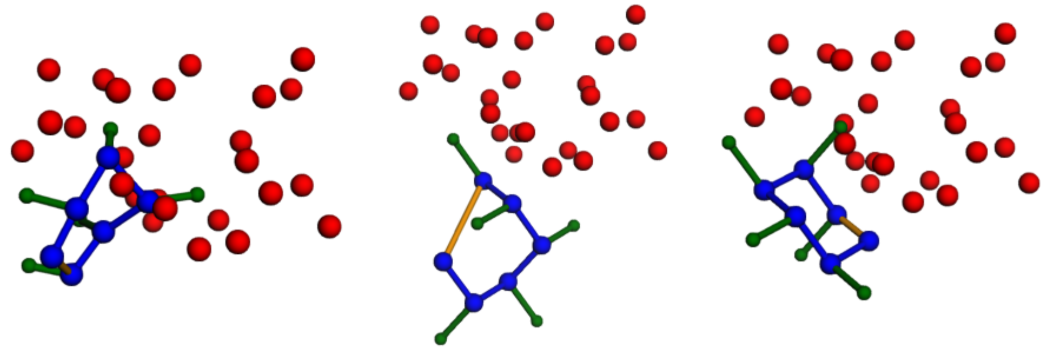

Figure S4: 3WNE result visualization. True PDB peptide (left) against the QUBO model result (middle) and the CP-MJ 1-NN result (right). The peptide is represented with blue and green dots (main and side-chain, respectively, connected by lines), and the red dots are the external protein residues.

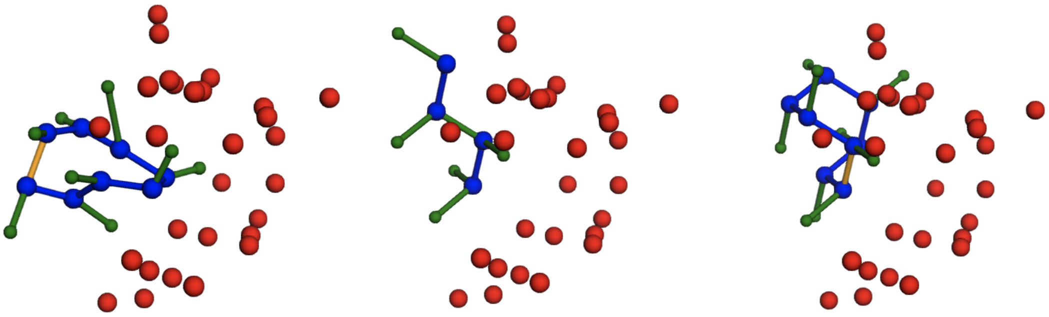

Figure S5: 3AV9 result visualization. True PDB peptide (left) against the QUBO model result (middle) and the CP-MJ 1-NN result (right). The peptide is represented with blue and green dots (main and side-chain, respectively, connected by lines), and the red dots are the external protein residues.

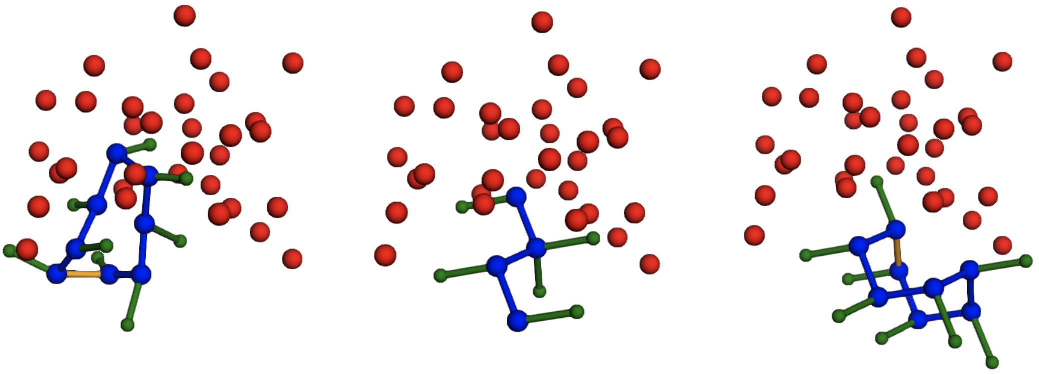

Figure S6: 3AVI result visualization. True PDB peptide (left) against the QUBO model result (middle) and the CP-MJ 1-NN result (right). The peptide is represented with blue and green dots (main and side-chain, respectively, connected by lines), and the red dots are the external protein residues.

I. Additional discussion points

I.1. Construction of the Hamiltonian

Initially, we pursued different problem encodings in parallel: the spatial encoding (see Supplementary F), the turn ancilla encoding (see Supplementary G), and the variable efficient turn encoding (see Manuscript Section 2). We implemented feature-complete versions of each, including all problem constraints and the external protein.

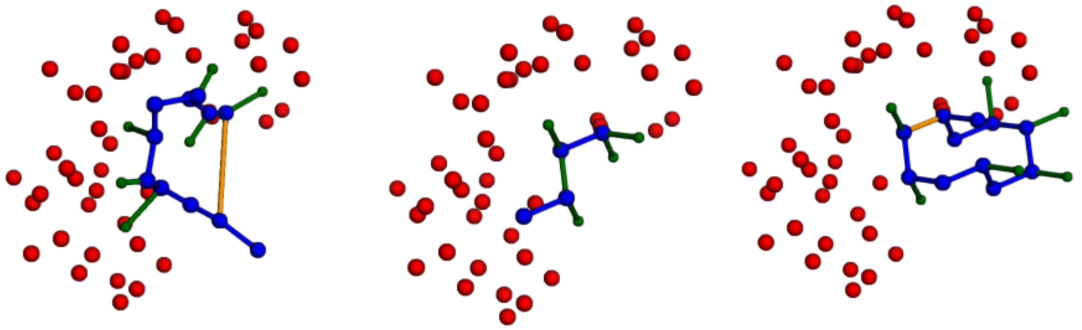

Figure S7: 2F58 result visualization. True PDB peptide (left) against the QUBO model result (middle) and the CP-MJ 1-NN result (right). The peptide is represented with blue and green dots (main and side-chain, respectively, connected by lines), and the red dots are the external protein residues.

Because the spatial encoding is not anchored to a specific starting point (e.g., the lattice origin), we expected it to be more robust and likely the more performant encoding scheme. However, the Hamiltonian variables and terms scaling of the spatial encoding are prohibitive at the problem sizes we are interested in solving. In fact, the number of terms in the spatial encoding QUBO Hamiltonian is so large that attempts at manipulating the Hamiltonian expression overloaded our hardware and caused the Python process to terminate. Because of this, we shifted our attention to one of the turn encoding approaches.

The turn ancilla approach also suffers from a scaling problem, namely, every distance calculation between pairs of residues must be upper-bounded by a fixed value, duplicated for every distance comparison] and this bound grows as the problem size grows. This means that the variable scaling of the turn ancilla approach scales worse than $\mathcal{O}(N^2)$ and also scales with a factor of $\log_2(N)$ [or $\log_2(\max(d(M, N)))$ considering the protein]. This is not true for the resource-efficient approach, which does not seek to bound all distance calculations, but rather includes ancilla bits to turn off the effect of those distances when necessary. Because of this, we expect the resource-efficient approach to actually be the most efficient and therefore most useful approach in solving the docking problem, as demonstrated in the scaling experiments. Details on the Hamiltonian variable and terms scaling experiments can be found in Supplementary E.

I.2. Lattice Considerations

The choice of the tetrahedral lattice over higher-resolution alternatives such as cubic or face-centered cubic helped keep the problem tractable, and helped reduced problem complexity for the turn encodings. Cubic lattices have 6 turns from each vertex, which would require 3 bits and generate two invalid turn directions that need to be accounted for, and the face-centered cubic lattice has 4 turns from any given face vertex and 6 turns from a given corner vertex, so the representation would need to vary, or additional invalid turn directions would need to be accounted for. In contrast, any vertex on the tetrahedral lattice has exactly 4 turns allowed from it, though they alternate in directions. This means we could fix the representation at 2 bits per turn, and simply introduce a sign flip according to the specific sub-lattice. This helps reduce the conceptual complexity, and it kept the Hamiltonian scaling to a minimum, whereas the cubic or face-centered cubic lattices would have exhibited even worse scaling.

Once the lattice structure is fixed, the depth (e.g., size) of the lattice needs to be established. There is no rule for this, other than it needs to be big enough to fit the peptide and a sufficient amount of the protein to properly guide the peptide conformation. It might seem that the lattice should be set big enough to fit the full protein on it—which is to say the full set of protein projections—but this is not necessarily the case, and may result in a lattice that is too large. If the lattice is grown too large, a couple risks are introduced. First, because we use the protein active site only, and not the full protein, regions around the sides of the protein (active site) start to appear, and any spatial encoding model may choose to place the peptide in these regions (assuming enough space exists). Although we did not observe this, it is possible that the peptide could be in a region that would actually be inside of the full protein complex. Second, and perhaps more importantly, allowing for full protein projection effectively doubles the number of protein influenced vertices that need to be included in the Hamiltonian. This means that from a starting point near the active site centroid (i.e., the lattice origin), any peptide conformation would need to walk through a double layer of protein blocking sites before it reaches any interaction sites that are on the far side of the protein (with respect to the centroid). As long as the protein clash penalty (μ_2) is set high enough, any turn encoding model would never choose such a solution. Similarly, for a spatial encoding if the lattice is set large enough to include those interaction projections, but no larger, then there would be no way for the spatial model to place the peptide in a way to reach those projections without

also landing on the blocking sites. As such, it does not help to include those interaction sites on the far side of the protein, as they introduce an additional scaling burden for all encodings.

Finally, we note that the use of a fixed lattice structure in our problem construction introduces some minimum error in the RMSD metric that cannot be overcome. The lattice assumes a fixed set of turn directions and a fixed length between vertices, neither of which occurs in the real world. This leads to model results that appear clean and highly structured, but that do not align with the more irregular conformations found in the PDB results. Figures S3 and 2 show the differences clearly. Given our problem definition, we find it more useful to focus on finding feasible solutions, and optimizing for high quality results per MJ interaction potentials within the feasible set.

I.3. Looking forward

While we stand by our work here as an important synthesis of prior art, and a valuable step forward in formulating the problem via the inclusion of the external protein, we have noted several limitations incurred by our simplifying assumptions. There are several alternatives for refinement. First, we suggest revisiting the tetrahedral lattice geometry. The tetrahedral geometry stemmed from the realistic inter-bond and dihedral angles commonly observed in atomistic space but it is not obvious if this property persists in two-particle CG amino acid representation. Continuous dihedral angle and variable bond lengths would be ideal but such implementation would be even harder to investigate using QUBO formulations. Second, we might consider revisiting the MJ interaction energies used to guide the conformation. MJ potentials are designed to capture effects between entire amino acids, and no CG equivalent potential exists in the literature to better represent the interaction potential for two-particle amino acids. Third, we could extend the constraints to also include specifying chirality, which has real-world implications on the final conformation. Fourth, the QUBO model currently only allows for 1-NN interactions. However, the preferred interaction radius is approximately 6.5Å, which translates to roughly 2-NN interactions on the tetrahedral lattice. Thus, an obvious extension would be to include 2-NN interactions between peptide residues. Finally, we note that the current lattice structure is grown at a fixed location and angle relative to the protein residues. There is no guarantee that this is the optimal lattice orientation, and in fact it is likely not to be the optimal orientation. It would be worthwhile to investigate methodologies to optimize lattice orientation, either as part of the solution pipeline or model solving, such that RMSD is further minimized.

References

[1] A. Perdomo, C. Truncik, I. Tubert-Brohman, G. Rose, A. Aspuru-Guzik, Construction of model hamiltonians for adiabatic quantum computation and its application to finding low-energy conformations of lattice protein models, Physical Review A 78 (1) (2008). doi:10.1103/physreva.78.012320.

[2] T. Babej, C. Ing, M. Fingerhuth, Coarse-grained lattice protein folding on a quantum annealer (2018). doi:10.48550/ARXIV.1811.00713.

[3] R. Babbush, A. Perdomo-Ortiz, B. O'Gorman, W. Macready, A. Aspuru-Guzik, Construction of energy functions for lattice heteropolymer models: Efficient encodings for constraint satisfaction programming and quantum annealing, in: Advances in Chemical Physics, John Wiley & Sons, Inc., 2014, pp. 201–244. doi:10.1002/9781118755815.ch05.

[4] A. Perdomo-Ortiz, N. Dickson, M. Drew-Brook, G. Rose, A. Aspuru-Guzik, Finding low-energy conformations of lattice protein models by quantum annealing, Scientific Reports 2 (1) (2012) 1–7. doi:10.1038/srep00571.

[5] D-Wave Systems Inc.
URL https://www.dwavesys.com/

[6] M. Fingerhuth, T. Babej, C. Ing, A quantum alternating operator ansatz with hard and soft constraints for lattice protein folding (2018). doi:10.48550/ARXIV.1810.13411.

[7] Rigetti Computing, Inc.
URL https://www.rigetti.com/

[8] A. Robert, P. K. Barkoutsos, S. Woerner, I. Tavernelli, Resource-efficient quantum algorithm for protein folding, npj Quantum Information 7 (1) (feb 2021). doi:10.1038/s41534-021-00368-4.

[9] S. Boulebnane, X. Lucas, A. Meyder, S. Adaszewski, A. Montanaro, Peptide conformational sampling using the quantum approximate optimization algorithm, npj Quantum Information 9 (1) (2023) 70. doi:10.1038/s41534-023-00733-5.

[10] A. Irbäck, L. Knuthson, S. Mohanty, C. Peterson, Folding lattice proteins with quantum annealing, Phys. Rev. Res. 4 (2022) 043013. doi:10.1103/PhysRevResearch.4.043013.

[11] Y. Xiang, S. Gubian, B. Suomela, J. Hoeng, Generalized simulated annealing for global optimization: the gensa package, R J. 5 (1) (2013) 13.

[12] J. Beasley, Heuristic algorithms for the unconstrained binary quadratic programming problem, London, England (1999).

[13] G. Palubeckis, Multistart tabu search strategies for the unconstrained binary quadratic optimization problem, Annals of Operations Research 131 (2004). doi:10.1023/B:ANOR.0000039522.58036.68.

[14] Y. Fang, P. Warburton, Minimizing minor embedding energy: an application in quantum annealing, Quantum Information Processing 19 (2020). doi:10.1007/s11128-020-02681-x.

[15] G. Rosenberg, M. Vazifeh, B. Woods, E. Haber, Building an iterative heuristic solver for a quantum annealer, Computational Optimization and Applications 65 (2016). doi:10.1007/s10589-016-9844-y.

[16] S. Miyazawa, R. L. Jernigan, Estimation of effective interresidue contact energies from protein crystal structures: quasi-chemical approximation, Macromolecules 18 (3) (1985) 534–552. doi:doi.org/10.1021/ma00145a039.

[17] L. Perron, Operations research and constraint programming at Google, in: Principles and Practice of Constraint Programming–CP 2011: 17th International Conference, CP 2011, Perugia, Italy, September 12-16, 2011. Proceedings 17, Springer, Springer Berlin Heidelberg, 2011, pp. 2–2. doi:10.1007/978-3-642-23786-7_2.

[18] D. Sehnal, S. Bittrich, M. Deshpande, R. Svobodová, K. Berka, V. Bazgier, S. Velankar, S. K. Burley, J. Koča, A. S. Rose, Mol* viewer: modern web app for 3D visualization and analysis of large biomolecular structures, Nucleic acids research 49 (W1) (2021) W431–W437. doi:doi.org/10.1093/nar/gkab314.

[19] N. Rego, D. Koes, 3dmol. js: molecular visualization with WebGL, Bioinformatics 31 (8) (2015) 1322–1324. doi:10.1093/bioinformatics/btu829.

J. Legends

Tables:

S1. Literature review of related work. Here "Intra." refers to intramolecular interactions and "Inter." refers to intermolecular interactions. 2D refers to a two-dimensional square lattice and 3D to a three-dimensional cubic one, respectively.

Figures:

S1. Problem scaling for each approach: spatial QUBO (SQ), turn ancilla turn encoding (TA), and resource efficient turn encoding (RE) as a function of the peptide length for polynomial (PUBO) and quadratic (QUBO) Hamiltonian forms. Number of protein residues included starts at $M = 0$ (top row) and increases until $M = 10$ (bottom row). (left) Number of variables required for the Hamiltonian for each approach. (middle) Number of terms ($\log_{10}$) required for the Hamiltonian for each approach. (right) End-to-end runtime for each approach.

S2. Problem instance generation pipeline for peptide 3WNE from an atomic-level, continuous coordinate PDB representation to a 2-particle coarse grained tetrahedral lattice representation. Details are described in the main text.

S3. 3AVN result visualization. True PDB peptide (left) against the QUBO model result (middle) and the CP-MJ 1-NN result (right). The peptide is represented with blue and green dots (main and side-chain, respectively, conected by lines), and the red dots are the external protein residues.

S4. 3WNE result visualization. True PDB peptide (left) against the QUBO model result (middle) and the CP-MJ 1-NN result (right). The peptide is represented with blue and green dots (main and side-chain, respectively, connected by lines), and the red dots are the external protein residues.

S5. 3AV9 result visualization. True PDB peptide (left) against the QUBO model result (middle) and the CP-MJ 1-NN result (right). The peptide is represented with blue and green dots (main and side-chain, respectively, connected by lines), and the red dots are the external protein residues.

S6. 3AVI result visualization. True PDB peptide (left) against the QUBO model result (middle) and the CP-MJ 1-NN result (right). The peptide is represented with blue and green dots (main and side-chain, respectively, connected by lines), and the red dots are the external protein residues.

S7. 2F58 result visualization. True PDB peptide (left) against the QUBO model result (middle) and the CP-MJ 1-NN result (right). The peptide is represented with blue and green dots (main and side-chain, respectively, connected by lines), and the red dots are the external protein residues.
